# Structural bioinformatics and gene expression analysis of maturase K from *Lavandula angustifolia* (lavender)

**DOI:** 10.3389/fmolb.2025.1628118

**Published:** 2025-07-10

**Authors:** Dafeng Liu, Na Li, Daoqi Song, Zhenming Lv

**Affiliations:** ^1^ Xinjiang Key Laboratory of Lavender Conservation and Utilization, College of Biological Sciences and Technology, Yili Normal University, Yili, China; ^2^ School of Life Sciences, Xiamen University, Xiamen, China

**Keywords:** lavandula x intermedia (lavandin), maturase K, prediction of structural models, RT-qPCR analysis, heat and salt stress

## Abstract

The chloroplast genome of plants contains a single gene encoding the splicing factor Maturase K (MatK). To elucidate the functional role and underlying mechanism of MatK, we investigated it in *Lavandula angustifolia* (lavender). Structural models of MatK1 and Matk2 were predicted using AlphaFold2, and potential active site residues were identified via the GalaxyWEB program. The results of RT-qPCR analysis revealed that the expression of *MatK1* and *MatK2* peaked in leaves at 14:00. For heat treatments, *MatK1* expression in leaves increased with the duration of heat exposure, reaching its highest levels at 40°C for 3 h and 30°C for 6 h, before declining. Similarly, under salt treatment, *MatK1* expression in leaves showed an increasing trend with exposure time, peaking at 300 mM NaCl for 3 h and 200 mM for 12 h, before decreasing. This study provides the first detailed characterization of Maturase K in *L. angustifolia*.

## Introduction

Lavender plants are compact, aromatic shrubs widely cultivated for their essential oils (EOs), which consist of complex blends of mono- and sesquiterpenoid alcohols, esters, oxides, and ketones (Crişan et al., 2023; de Melo Alves Silva et al., 2023). The *Lavandula* genus includes 30 recognized species, with *Lavandula angustifolia*, *Lavandula latifolia*, and *Lavandula x intermedia*—a natural hybrid of *L. latifolia* and *L. angustifolia*—being of significant economic importance (Crişan et al., 2023; de Melo Alves Silva et al., 2023; Landmann et al., 2007; Liu et al., 2025d; Liu et al., 2025c). The highest-quality EOs are derived from the flowering tops of *L. angustifolia*, commonly known as ‘true lavender’, which has been valued for its distinctive fragrance since ancient times. Lavender EOs have diverse applications in cosmetics, hygiene, and alternative medicine (Hedayati et al., 2024; Khan et al., 2024; Li et al., 2024). For example, EOs with elevated camphor concentrations are used in inhalants to treat respiratory conditions like coughs and colds, as well as in liniments and balms for topical pain relief (Malloggi et al., 2021; Batiha et al., 2023; Braunstein and Braunstein, 2023; Liu et al., 2024a). Camphor has also been investigated as a radiosensitizing agent to enhance tumor oxygenation prior to radiotherapy (Bungau et al., 2023; Crişan et al., 2023; de Melo Alves Silva et al., 2023; Dewanjee et al., 2023; Khan et al., 2024).

The production of EOs in plants is closely linked to photosynthesis, a process involving several enzymes, including Maturase K (MatK). Recently, MatK has gained attention as a crucial gene due to its strong phylogenetic signal (Mukhopadhyay and Hausner, 2024). The high rate of amino acid substitution in MatK is attributed to the nearly uniform distribution of substitution rates across the three codon positions, in contrast to most protein-coding genes, where substitution rates are typically biased toward the third codon position (Unnikrishnan et al., 2021; Zhang et al., 2021; Algarni, 2022). In addition to its significance in plant phylogenetics, MatK is the only putative group II intron maturase encoded in the chloroplast genome. MatK enzymes catalyze the nonautocatalytic removal of introns from precursor RNAs. These enzymes typically consist of three domains: a reverse-transcriptase domain, domain X (the proposed maturase functional domain), and a zinc-finger-like domain. While there is a substantial body of literature on MatK in plants (Mukhopadhyay and Hausner, 2024; Tripodi, 2023; Liu et al., 2024c; Muino et al., 2024; Oyelakin et al., 2024; Tiono et al., 2024; Urbina et al., 2024), its specific function and mechanism in lavender remain poorly understood.

Herein, we used AlphaFold2 program to predict structural models of MatK1 and Matk2, and then identified potential active site residues via the GalaxyWEB program. Gene expression analysis revealed that *MatK1* was upregulated by 553.8-fold in leaves, 4.2-fold in flowers, 1.7-fold in stems, and 1.1-fold in roots at 14:00. Similarly, *MatK2* expression was upregulated by 267.5-fold in leaves, 4.2-fold in flowers, 1.3-fold in stems, and 1.0-fold in roots at 14:00. *MatK1* expression in leaves increased with the duration of heat treatment, peaking at 40°C for 3 h and 30°C for 6 h, before declining. Similarly, under salt treatment, *MatK1* expression in leaves showed a progressive increase, peaking at 300 mM NaCl for 3 h and 200 mM for 12 h, before decreasing. This study provides the first comprehensive analysis of Maturase K in *L. angustifolia*, offering valuable insights for improving the quality of lavender essential oil.

## Results

### Biochemical characteristics of Matk1 and Matk2

**FIGURE 1:**
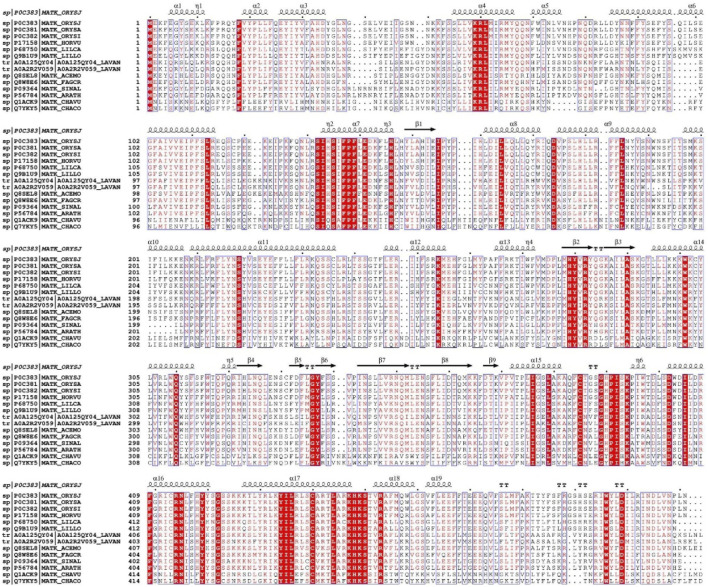
Sequence alignment of maturase K family. The alignment employs the ClustalW default color scheme, where conserved amino acids are highlighted in darker shades compared to variable residues. It includes the following reference protein sequences: P0C383, *Oryza sativa subsp. japonica* (Rice); P0C381, *Oryza sativa* (Rice); P0C382, *Oryza sativa subsp. indica* (Rice); P17158, *Hordeum vulgare* (Barley); P68750, *Lilium canadense* (Canada lily); Q9B1U9, *Lilium longiflorum* (Trumpet lily); A0A125QY04, *Lavandula angustifolia* (Lavender); A0A2R2V059, *Lavandula angustifolia* (Lavender); Q8SEL8, *Acer monspessulanum* (Montpellier maple); Q8W8E6, *Fagus crenata* (Japanese beech); P09364, *Sinapis alba* (White mustard, Brassica hirta); P56784, *Arabidopsis thaliana* (Mouse-ear cress); Q1ACK9, *Chara vulgaris* (Common stonewort); Q7YKY5, *Chara connivens* (Convergent stonewort).

Bioinformatics analysis of the two target proteins, MatK1 and MatK2, was performed using data retrieved from the UniProt database (MatK1, entry ID A0A2R2V059; MatK2, entry ID A0A125QY04) (Figure 1, Supplementary Figure S1–S5). The molecular weights of MatK1 and MatK2 were approximately 60.31 kDa and 60.89 kDa, respectively (Table 1). Their molecular formulas were C_2784_H_4317_N_751_O_722_S_14_ for MatK1 and C_2801_H_4350_N_762_O_736_S_13_ for MatK2. The amino acid composition of MatK1 included 36 negatively charged residues and 70 positively charged residues, while MatK2 contained 35 negatively charged residues and 70 positively charged residues. The grand average of hydropathy (GRAVY) values for MatK1 and MatK2 were −0.10 and −0.12, respectively (Table 1). The aliphatic indexes for MatK1 and MatK2 were 103.02 and 101.24, respectively (Table 1). Both MatK1 and MatK2 had an estimated half-life of 30 h (Table 1). The isoelectric points (pI) for MatK1 and MatK2 were 10.01 and 10.04, respectively, with protein instability indices of 51.32 and 50.08 (Table 1).

**TABLE 1:** Characteristics of Matk1 and Matk2.

Name	Number of amino acids	Molecular weight (kDa)	Theoretical pI^a^	Instability index	Aliphatic index	GRAVY^b^	Estimated half-life (h)
Matk1	506	60.31	10.01	51.32	103.02	−0.10	30
Matk2	516	60.89	10.04	50.08	101.24	−0.12	30

Note.

^a^ Isoelectric point.

^b^ GRAVY, grand average of hydropathy.

### Secondary structure prediction of Matk1 and Matk2

**FIGURE 2:**
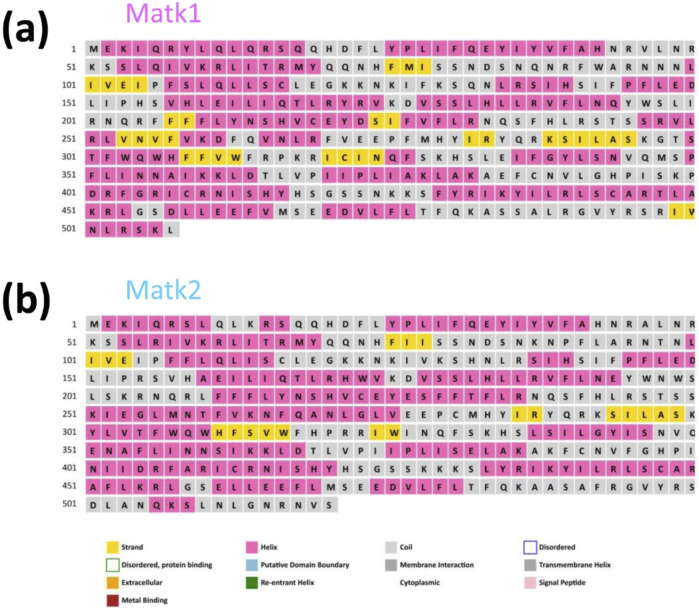
Prediction of **(a)** Matk1 and **(b)** Matk2 secondary structure models.

PSIPRED analysis (Buchan et al., 2024; Jones, 1999) revealed that MatK1 contains 227 alpha helices (44.86%) in its secondary structure, along with a significant number of extended strands and random coils (Figure 2a; Table 2). Similarly, MatK2 consists of 216 alpha helices (41.86%) and numerous strands and coils in its predicted secondary structure (Figure 2b; Table 2).

**TABLE 2:** Secondary structure prediction of Matk1 and Matk2.

Secondary structure	Alpha helix		Extended strand		Random coil	
Residual Properties	Number of residues	% of residues	Number of residues	% of residues	Number of residues	% of residues
Matk1	227	44.86	55	10.87	224	44.27
Matk2	216	41.86	57	11.05	243	47.09

### Prediction and quality assessment of Matk1 and Matk2 structures

The three-dimensional (3D) structures of MatK1 and MatK2 were predicted using AlphaFold2 (Wayment-Steele et al., 2023; Jumper et al., 2021), which employs deep learning algorithms for more accurate and reliable protein structure predictions compared to traditional homology modeling methods.

**FIGURE 3:**
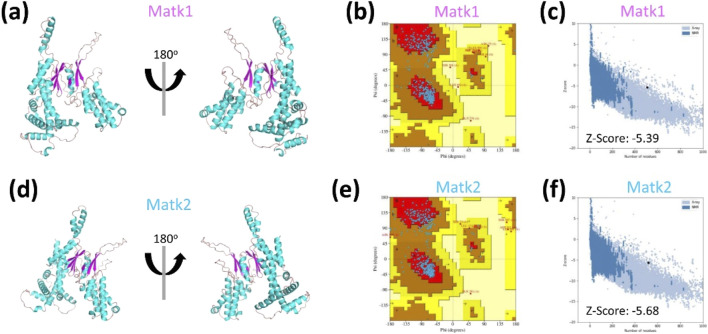
Structural prediction and quality assessment of MatK1 and MatK2. The three-dimensional (3D) structures of **(a)** MatK1 and **(d)** MatK2 were predicted using AlphaFold2. Both models are depicted as cyan ribbon diagrams from two distinct orientations, with α-helices in pink and β-sheets in cyan. Structural validation was performed using Ramachandran plot analysis [**(b)** for Matk1, **(e)** for Matk2], where the most favorable residue conformations are highlighted in red, and less favorable regions are shown in progressively lighter shades. Additionally, **(c,f)** ProSA analysis yielded Z-scores of −5.39 (MatK1) and −5.68 (MatK2), confirming the high quality of the predicted models.

To assess the quality of the predicted models (Figures 3a,d), we used the Ramachandran plot to evaluate the dihedral angles of the protein backbone, ensuring they fell within acceptable regions indicative of a stable conformation. For MatK1, 86.5% of the residues were in the most favored region, 11.9% in the additionally allowed region, 0.8% in the generously allowed region, and 0.8% in the disallowed region (Figure 3b; Table 3). For MatK2, 84.6% of residues were in the most favored region, 13.7% in the additionally allowed region, 1.4% in the generously allowed region, and 0.2% in the disallowed region (Figure 3e; Table 3).

**TABLE 3:** Ramchandran plot analysis of structural models of MatK1 and Matk2 using PDBsum.

Residues	Residues in most favored regions		Residues in additional allowed regions		Residues in generously allowed regions		Residues in disallowed regions	
Structural models	Number of residues	% of residues^a^	Number of residues	% of residues	Number of residues	% of residues	Number of residues	% of residues
Matk1^a^	415	86.5	57	11.9	4	0.8	4	0.8
Matk2^b^	413	84.6	67	13.7	7	1.4	1	0.2

Note.

^a^ Number of end-residues (excl. Gly and Pro): 2; Number of glycine residues: 10; Number of proline residues: 14.

^b^ Number of end-residues (excl. Gly and Pro): 2; Number of glycine residues: 13; Number of proline residues: 13.

ProSA analysis revealed Z-scores of −5.39 for MatK1 and -5.68 for MatK2 (Figures 3c,f), further supporting the high quality of the predicted models.

**FIGURE 4:**
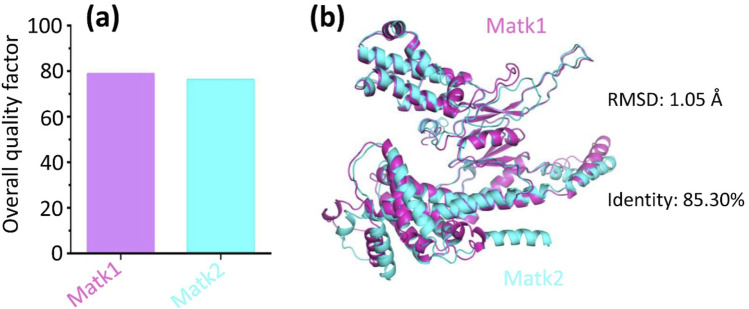
Structure comparison between Matk1 (in magenta) and Matk2 (in cyan). **(a)** The overall quality factors of structural models of Matk1 and Matk2. **(b)** Despite adopting a similar overall fold, MatK1 displayed a root mean square deviation (RMSD) of 1.05 Å (all atoms) relative to MatK2, with 85.30% amino acid sequence identity between the two proteins.

While the overall fold of MatK1 closely resembles that of MatK2 (Figure 4), the root mean square deviation (RMSD) for all atoms was 1.05 Å, with a sequence identity of 85.30% (Figure 4).

### Predicting the active sites of Matk1 and Matk2

**FIGURE 5:**
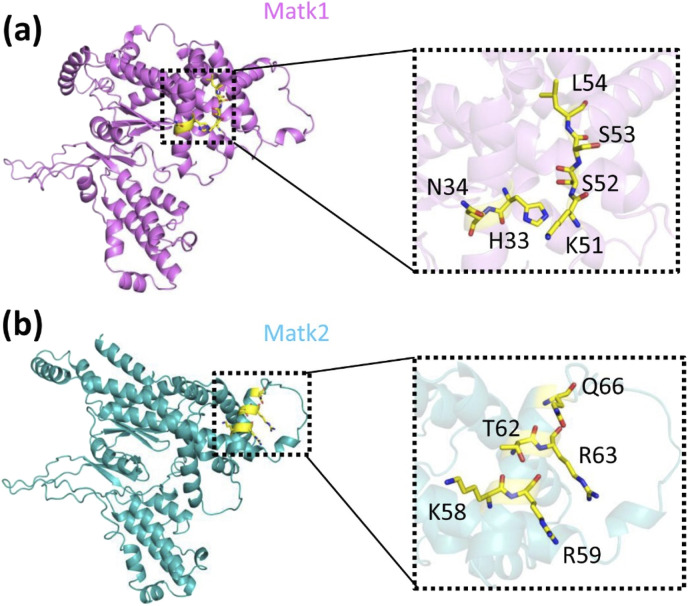
Predicting **(a)** Matk1 and **(b)** Matk2 active site residues. **(a)** The GalaxyWEB program predicted H33, N34, K51, S52, S53 and L54 as the active site residues of MatK1 (in magenta). **(b)** In MatK2 (in cyan), the active site residues identified were K58, R59, T62, R63, and Q66. The ribbon diagram of each model is shown, with a close-up view of each active site on the right.

Using the predicted models (Figures 3–5), we utilized the GalaxyWEB program (Seok et al., 2021; Heo et al., 2013; Heo et al., 2016; Ko et al., 2012) to identify the active sites of MatK1 and MatK2. The analysis revealed that the active site residues of MatK1 are H33, N34, K51, S52, S53, and L54 (Figure 5a). For MatK2, the active site residues include K58, R59, T62, R63, and Q66 (Figure 5b). These residues are likely involved in substrate interactions, potentially forming bonds with the substrate’s side chain atoms.

### Gene expression profiles of *Matk1* and *Matk2* in various tissues

**FIGURE 6:**
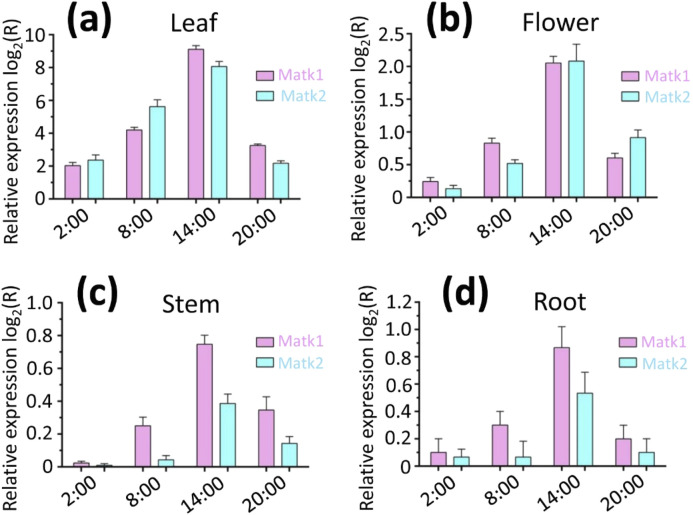
Expression levels of Matk1 and Matk2 in **(a)** leaf, **(b)** flower, **(c)** stem and **(d)** root within a 24 h day/night cycle. Relative expression analysis was conducted using RT-qPCR (real-time quantitative polymerase chain reaction). The relative expression ratios were presented as log_2_ values, where a ratio greater than zero indicated upregulation of gene expression.

To investigate the spatiotemporal expression profiles of *MatK1* and *MatK2*, we performed real-time quantitative polymerase chain reaction (RT-qPCR) using gene-specific primers (Supplementary Table S1). The results showed that the highest expression of both *MatK1* and *MatK2* occurred in the leaves at 14:00 (Figure 6). Specifically, *MatK1* expression was upregulated by 553.8-fold in leaves, 4.2-fold in flowers, 1.7-fold in stems, and 1.1-fold in roots at 14:00 (Figure 6). Similarly, *MatK2* expression was upregulated by 267.5-fold in leaves, 4.2-fold in flowers, 1.3-fold in stems, and 1.0-fold in roots at 14:00 (Figure 6). These results suggest that *MatK1* and *MatK2* are primarily involved in chloroplast photosynthesis, aligning with previous studies (Muino et al., 2024; Hertel et al., 2013; Barthet and Hilu, 2007; Qu et al., 2018).

### Expression levels of genes *Matk1* and *Matk2* under heat and salt treatments

**FIGURE 7:**
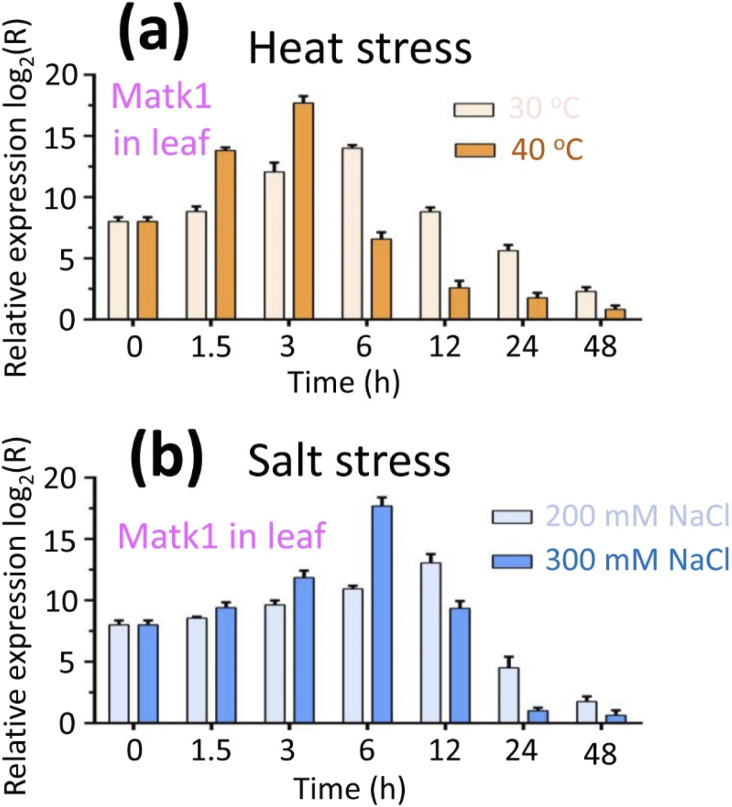
RT-qPCR data analysis of gene *Matk1* in leaf under **(a)** heat and **(b)** salt stress conditions. **(a)** For heat stress, plants were exposed to 30°C for 48 h and 40°C for 48 h, respectively. **(b)** For salt stress, plants were exposed to 200 mM NaCl for 48 h and 300 mM NaCl for 48 h, respectively. The relative expression level of the Matk1 gene in leaf was calculated using the 2^−ΔΔCT^ method.

We conducted RT-qPCR analysis to examine the expression levels of the *MatK1* gene under heat and salt treatments in leaves, as *MatK1* exhibited higher expression in leaves compared to *MatK2* (Figure 6). The results showed that *MatK1* expression in leaves increased with the duration of heat treatment, peaking at 40°C for 3 h and 30°C for 6 h, before declining (Figure 7a). Similarly, *MatK1* expression in leaves also increased with the duration of salt treatment, peaking at 300 mM NaCl for 3 h and 200 mM for 12 h, before decreasing (Figure 7a). These findings suggested that temperature and salt concentration influence the photosynthetic rate of lavender, supporting the link between *MatK1* and photosynthesis.

## Discussion

In this work, we generated structural models using AlphaFold2 and employed the GalaxyWEB program to predict potential active site residues. At 14:00, *MatK1* expression was significantly upregulated, showing a 553.8-fold increase in leaves, 4.2-fold in flowers, 1.7-fold in stems, and 1.1-fold in roots. Similarly, *MatK2* expression increased by 267.5-fold in leaves, 4.2-fold in flowers, 1.3-fold in stems, and remained nearly unchanged (1.0-fold) in roots. Under heat stress, *MatK1* transcript levels in leaves progressively increased, peaking after 3 h at 40°C and 6 h at 30°C, followed by a decline. Similarly, under salt stress, *MatK1* expression in leaves rose with prolonged exposure, peaking after 3 h at 300 mM NaCl and after 12 h at 200 mM, before decreasing. This study provides the first comprehensive analysis of Maturase K in *L. angustifolia*, offering valuable insights that could enhance the quality of lavender essential oil.

The MatK reading frame is present in all known autotrophic land-plant chloroplast genomes containing group II introns, as well as in basal streptophyte algae (Mukhopadhyay and Hausner, 2024; Liu et al., 2018; Ho et al., 2021; Oyelakin et al., 2024). Despite their low sequence identity (Figure 1), these active sites coordinate magnesium ions (Mg^2+^), primarily via negatively charged residues. The maturase K (MatK) family may employ divergent catalytic mechanisms to promote the splicing of both its own and other chloroplast group II introns. To elucidate these mechanisms, we are examining the structural and mechanistic basis of MatK-catalyzed reactions using experimental techniques, including X-ray crystallography. In the streptophyte alga *Zygnema*, the fern *Adiantum capillus-veneris*, and the parasitic land plants *Epifagus virginiana*, *Cuscuta exaltata*, and *Cuscuta reflexa*, MatK exists as a stand-alone reading frame, with the trnK gene being absent. This suggests that MatK functions ‘in trans’, likely involved in splicing pre-RNAs other than its corresponding trnK intron (Hertel et al., 2013; Qu et al., 2018; Barthet and Hilu, 2007). Notably, among all analyzed embryophytes, only parasitic species have lost MatK. The retention of MatK in chloroplast genomes across early streptophytes indicates that its presence is not a random event. Furthermore, attempts at reverse genetic manipulation of the chloroplast MatK reading frame through transplastomic mutagenesis have been unsuccessful, supporting the notion that MatK is an essential gene.

To elucidate the functional role of *L. angustifolia* MatK in terpenoid biosynthesis and stress responses, we will employ a combination of *in vivo* and *in vitro* assays. Targeted knockdown of MatK via virus-induced gene silencing (VIGS) and RNA interference (RNAi) will be used to assess loss-of-function phenotypes, while Agrobacterium-mediated overexpression studies will evaluate gain-of-function effects on metabolic pathways. Functional validation will be further confirmed through mutant complementation in transgenic lines. These integrated approaches will systematically investigate MatK molecular mechanisms, including its potential interactions with plastid-encoded proteins and regulatory influence on secondary metabolite production. Transcriptional, translational, and metabolic changes will be monitored using quantitative PCR, Western blotting, and HPLC analyses, respectively.

In conclusion, our study offers a novel approach to comprehensively investigate the functional mechanisms of MatK (Maturase K) in *L. angustifolia* (lavender), with the goal of enhancing the quality of lavender essential oils.

## Materials and methods

### Bioinformatics analysis

The amino acid sequences of MatK1 (UniProt code A0A2R2V059) and MatK2 (UniProt code A0A125QY04) (Figure 1, Supplementary Figure S1–S5) were analyzed using the ProtParam (Gasteiger, 2003; Duvaud et al., 2021) to predict their chemical properties and physicochemical parameters.

### Prediction of structural models

Structural predictions of the target proteins (MatK1 and MatK2) were performed using the AlphaFold2 program (Wayment-Steele et al., 2023; Jumper et al., 2021). Secondary structures were predicted with the PSIPRED program (Jones, 1999; Buchan et al., 2024), and active site residues were identified using the GalaxyWEB program (Ko et al., 2012; Heo et al., 2013; Heo et al., 2016; Seok et al., 2021). Multiple sequence alignment data were obtained from the LSQKAB program within the CCP4 suite (Collaborative Computational Project, Number, 1994), and the root mean square deviation (RMSD) for Cα atoms was calculated. Structural images were generated using PyMOL 2.3.4 (https://www.pymol.org/2/).

### Quality assessment of structural models

To validate the tertiary structures, we used the PDBsum database (Laskowski, 2022; de Beer et al., 2014; Laskowski, 2004; 2009; Laskowski et al., 2017) to generate Ramachandran plots for MatK1 and MatK2. This tool helps assess and validate protein structure quality by identifying geometric errors and improving accuracy. The Ramachandran plot specifically evaluates the stereochemical properties of the structures, displaying the dihedral angles of amino acid residues, highlighting allowed conformational regions, and identifying disallowed orientations.

Additionally, ProSA (Protein Structure Analysis) is a widely used tool for analyzing and validating predicted protein models (Wiederstein and Sippl, 2007). It aids in the analysis of protein structures derived from X-ray crystallography and NMR spectroscopy, identifying structural errors and pinpointing problematic regions, thereby improving the interpretation of the protein structures.

### Expression levels of genes *Matk1* and *Matk2*

To quantify the expression levels of *MatK1* and *MatK2* under different light conditions, real-time quantitative PCR (RT-qPCR) was performed using PowerUp SYBR Green Master Mix (Applied Biosystems). Total RNA was extracted with the Universal Plant Total RNA Extraction Kit (Bioteke, Beijing, China) according to the manufacturer’s instructions. cDNA was synthesized from RNA using the PrimeScript 1st Strand cDNA Synthesis Kit (Takara, Kyoto, Japan). The primers used are listed in Supplementary Table S1. RT-qPCR was conducted with the Applied Biosystems QuantStudio 5 instrument, and data were analyzed using the 2^−ΔΔCT^ method (Green and Sambrook, 2018; Schmittgen and Livak, 2008; Livak and Schmittgen, 2001; Liu et al., 2025d; Liu et al. 2025a; Liu et al. 2025b; Liu et al. 2025c; Liu et al. 2024a; Liu et al. 2024b). Relative expression ratios are presented as log_2_ values in histograms. Beta-actin served as the housekeeping gene for normalization, with a positive control using the beta-actin gene. A ratio greater than zero indicated up-regulation, while a ratio less than zero indicated downregulation.

### Statistical analysis

All experiments were conducted at least in triplicate. The data were expressed as mean ± SD. Statistical analysis was conducted using Origin 8.5, Microsoft Excel 2013 and SPSS 19.0. In the all statistical evaluations, *p* < 0.05 was considered statistically significant, and *p* < 0.01 was considered high statistically significant.

## Data Availability

The datasets presented in this study can be found in online repositories. The names of the repository/repositories and accession number(s) can be found in the article/Supplementary Material.
