# Supplementary material for: Structural bioinformatics and gene expression analysis of maturase K from Lavandula angustifolia (lavender)
Source: Front Mol Biosci. 2025 Jul 10;12:1628118. doi: 10.3389/fmolb.2025.1628118 (PMC12286831; doi:10.3389/fmolb.2025.1628118)
Supplement: Supplementary file 1 [file Supplementaryfile1.pdf]

Supplementary Material

Structural bioinformatics and gene expression analysis of Maturase K from *Lavandula angustifolia* (lavender)

Dafeng Liu[1,2,*], Na Li[1], Daoqi Song[2] and Zhenming Lv[2]

[1]Xinjiang Key Laboratory of Lavender Conservation and Utilization, College of Biological Sciences and Technology, Yili Normal University, Yining 835000, Xinjiang, China;

[2]School of Life Sciences, Xiamen University, Xiamen 361102, Fujian, China.

[*]Correspondence: dafeli@sina.cn or dafeli-dafeli@hotmail.com

Figure S1.

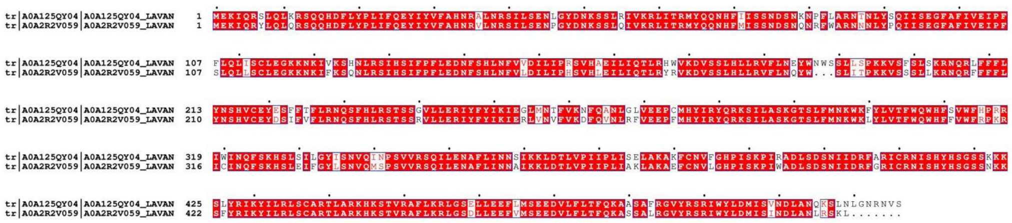

Figure S1. The sequence of Matk1 was compared with the sequence of Matk2. The sequences of MatK1 and MatK2 were aligned using ClustalW, employing default parameters and the associated color scheme, where conserved amino acids are highlighted with greater intensity. The alignment incorporated the following reference proteins: A0A2R2V059 (MatK1, *Lavandula angustifolia*) and A0A125QY04 (MatK2, *Lavandula angustifolia*).

Figure S2.

MEKIQRYLQLQRSQQHDFLYPLIFQEYIYVFAHNRVLNRSILSENPGYDNKSSLQIVKRLITRMYQQNHFMISSN
DSNQNRFWARNNNLYPQIISEGFAFIVEIPFSLQLLSCLEGKKNKIFKSQNLRSIHSIFPFLEDNFSHLNFVLDILIPH
SVHLEILIQTLRYRVKDVSSLHLLRVFLNQYWSLITPKKVSSSLLKRNQRFFFFLYNSHVCEYDSIFVFLRNQSFHLR
STSSRVLLERIYFYIKIERLVNVFVKDFQVNLRFVEEPFMHYIRYQRKSILASKGTSLFMNKWKLYLVTFWQWHFF
VWFRPKRICINQFSKHSLEIFGYLSNVQMSPSVVRSQILENAFLINNAIKKLDTLVPIIPLIAKLAKAEFCNVLGHPIS
KPIWADLSDSNIIDRFGRICRNISHYHSGSSNKKSFYRIKYILRLSCARTLARKHKSTVRAFLKRLGSDLLEEFVMSE
EDVLFLTFQKASSALRGVYRSRIWYLDMISINDLANLRSKL

Figure S2. Amino acid sequence of Matk1

Figure S3.

MEKIQRSLQLKRSQQHDFLYPLIFQEYIYVFAHNRALNRSILSENLGYDNKSSLRIVKRLITRMYQQNHFIISSNDS
NKNPFLARNTNLYSQIISEGFAFIVEIPFFLQLISCLEGKKNKIVKSHNLRSIHSIFPFLEDNFSHLNFVVDILIPRSVH
AEILIQTLRHWVKDVSSLHLLRVFLNEYWNWSSLLSPKKVSFSLSKRNQRLFFFLYNSHVCEYESFFTFLRNQSFH
LRSTSSGVLLERIYFYIKIEGLMNTFVKNFQANLGLVEEPCMHYIRYQRKSILASKGTSLFMNKWKFYLVTFWQW
HFSVWFHPRRIWINQFSKHSLSILGYISNVQINPSVVRSQILENAFLINNSIKKLDTLVPIIPLISELAKAKFCNVFGH
PISKPIRADLSDSNIIDRFARICRNISHYHSGSSKKKSLYRIKYILRLSCARTLARKHKSTVRAFLKRLGSELLEEFLMS
EEDVLFLTFQKAASAFRGVYRSRIWYLDMISVNDLANQKSLNLGNRNVS

Figure S3. Amino acid sequence of Matk2

Figure S4.

ATGGAAAAAATTCAGCGTTATCTGCAACTGCAGCGTTCCCAACAGCACGACTTTCTGTATCCTCTGATCTTCC
AGGAGTACATCTATGTATTCGCGCATAACCGTGTACTGAACCGTTCCATCCTGTCTGAAAACCCGGGTTACGA
TAACAAAAGCTCCCTGCAGATCGTGAAACGTCTGATTACCCGTATGTATCAGCAGAACCACTTCATGATCAGC
AGCAACGATTCCAACCAGAACCGCTTTTGGGCCCGCAACAACAACCTGTACCCGCAGATTATCTCTGAGGGT
TTCGCATTCATCGTGGAAATCCCGTTCTCTCTGCAGCTGCTGAGCTGTCTGGAGGGCAAAAAGAATAAAATC
TTTAAGTCTCAGAACCTGCGTAGCATTCACAGCATCTTCCCGTTCCTGGAAGATAACTTCAGCCACCTGAATT
TCGTCCTGGACATTCTGATTCCGCACTCTGTGCACCTGGAAATCCTGATTCAAACCCTGCGTTACCGTGTAAA
AGATGTGTCTTCCCTGCACCTGCTGCGCGTTTTCCTGAACCAGTATTGGTCCCTGATCACGCCGAAAAAAGT
ATCTAGCTCTCTGCTGAAACGCAACCAGCGTTTCTTCTTTTTCCTGTATAACTCCCATGTGTGCGAGTACGACT
CTATTTTCGTCTTCCTGCGTAACCAGTCCTTCCATCTGCGCAGCACCAGCAGCCGTGTTCTGCTGGAACGCAT
CTATTTCTATATCAAAATCGAACGTCTGGTGAACGTATTTGTCAAAGACTTCCAGGTAAACCTGCGCTTCGTG
GAAGAACCGTTTATGCACTATATCCGTTACCAGCGTAAATCCATCCTGGCTTCCAAAGGTACCTCCCTGTTTAT
GAACAAATGGAAACTGTACCTGGTAACCTTTTGGCAGTGGCACTTCTTCGTGTGGTTCCGTCCAAAACGCAT
CTGCATCAACCAGTTCTCCAAACATTCCCTGGAGATTTTTGGCTACCTGTCTAACGTACAGATGTCTCCGAGC
GTCGTGCGCTCTCAAATCCTGGAAAACGCCTTCCTGATCAACAACGCCATCAAAAAGCTGGACACCCTGGTA
CCGATCATTCCGCTGATTGCAAAGCTGGCGAAAGCAGAATTCTGCAACGTTCTGGGCCATCCAATCTCTAAA
CCGATTTGGGCGGATCTGTCCGATAGCAACATCATCGACCGTTTTGGTCGTATTTGCCGTAACATTTCTCACTA
TCACAGCGGCAGCTCCAACAAGAAATCCTTCTATCGCATCAAATACATTCTGCGTCTGTCTTGTGCCCGCACC
CTGGCTCGTAAACACAAATCTACTGTGCGTGCTTTCCTGAAACGCCTGGGCTCTGATCTGCTGGAAGAGTTC
GTCATGTCTGAAGAGGATGTTCTGTTCCTGACGTTTCAGAAAGCGTCCTCTGCACTGCGCGGTGTTTATCGT
TCCCGTATTTGGTACCTGGATATGATCTCCATCAACGATCTGGCTAATCTGCGTTCTAAACTG

Figure S4. Gene sequence of *Matk1* after codon optimization

Figure S5.

ATGGAAAAAATTCAACGTTCCCTGCAGCTGAAGCGCTCTCAACAACACGATTTCCTGTATCCGCTGATCTTCC
AGGAGTACATCTATGTTTTCGCGCACAACCGTGCCCTGAATCGTTCCATCCTGTCTGAAAACCTGGGTTACGA
TAACAAATCTTCCCTGCGCATCGTAAAACGTCTGATCACCCGTATGTACCAGCAGAATCACTTCATCATTTCTT
CCAACGATAGCAACAAAAACCCGTTCCTGGCTCGTAACACCAACCTGTACAGCCAAATCATCTCCGAAGGTT
TTGCCTTTATCGTGGAGATTCCGTTCTTCCTGCAGCTGATTAGCTGCCTGGAAGGCAAAAAGAACAAAATTG
TGAAAAGCCACAACCTGCGTTCCATCCACTCTATTTTTCCGTTCCTGGAAGATAACTTCTCCCACCTGAACTTC
GTGGTAGACATCCTGATCCCACGTTCTGTACATGCAGAAATCCTGATCCAGACTCTGCGTCACTGGGTAAAA
GACGTTTCTTCTCTGCATCTGCTGCGCGTTTTCCTGAACGAATATTGGAACTGGTCCTCCCTGCTGTCCCCAA
AAAAGGTGTCTTTTTCTCTGTCCAAGCGCAACCAGCGCCTGTTCTTCTTCCTGTACAACAGCCACGTTTGCG
AATATGAGTCCTTCTTCACGTTCCTGCGTAACCAGAGCTTCCACCTGCGTTCCACTTCTTCTGGTGTACTGCTG
GAGCGTATCTACTTCTATATCAAAATCGAAGGTCTGATGAACACCTTTGTAAAAAATTTTCAGGCAAACCTGG
GCCTGGTTGAGGAACCGTGTATGCACTATATTCGCTATCAGCGTAAAAGCATCCTGGCTTCCAAAGGTACTAG
CCTGTTCATGAACAAGTGGAAATTCTACCTGGTAACCTTCTGGCAGTGGCATTTCAGCGTGTGGTTCCACCCT
CGTCGTATCTGGATTAACCAGTTCTCCAAACACAGCCTGTCCATCCTGGGTTACATTTCTAATGTTCAGATTAA
CCCGAGCGTGGTACGTAGCCAGATTCTGGAAAACGCCTTCCTGATCAATAACAGCATCAAAAAACTGGACAC
TCTGGTTCCGATCATCCCTCTGATCTCCGAGCTGGCGAAAGCCAAGTTTTGCAACGTTTTCGGTCACCCGATC
AGCAAACCAATTCGTGCCGATCTGTCTGACTCCAACATCATTGACCGTTTCGCTCGCATCTGCCGTAACATTA
GCCACTACCATTCTGGTTCCTCTAAAAAAAAATCTCTGTACCGCATCAAATATATTCTGCGTCTGTCCTGCGCG
CGTACCCTGGCACGTAAACACAAATCCACCGTTCGTGCTTTTCTGAAACGTCTGGGTTCCGAACTGCTGGAG
GAATTCCTGATGAGCGAAGAGGATGTGCTGTTCCTGACCTTTCAGAAAGCAGCTTCTGCGTTTCGTGGCGT
GTACCGTTCTCGTATTTGGTACCTGGACATGATCAGCGTGAACGACCTGGCTAACCAGAAATCTCTGAACCTG
GGTAATCGTAACGTTAGC

Figure S5. Gene sequence of *Matk2* after codon optimization

Table S1.

Table S1. Primers used for RT-qPCR in this study

Genes	Primers	Primer sequence (5'-3')
Beta-actin	Forward primer	ATGGCCGAAGCCGAGGATATTCAGC
	Reverse primer	TTAGAAGCATTTTCTGTGAACGATCGACGGGC
Matk1	Forward primer	AAATAGAATTTCTTTCTTCGTCTTTACAAAAAAAAAAAAA
	Reverse primer	TCTCGCCTATTTACTACGGCGACGAACAATCAAATTATCA
Matk2	Forward primer	TTTATTCCTTTTTCTACTTCTTCTCCCAAGTGCAGAATAAC
	Reverse primer	CATGTATTATTTCATTCAAAATGCAACTTTGAAATTGCGTAGGTGATG
