# Supplementary material for: Structural bioinformatics and gene expression analysis of maturase K from Lavandula angustifolia (lavender)
Source: Front Mol Biosci. 2025 Jul 10;12:1628118. doi: 10.3389/fmolb.2025.1628118 (PMC12286831; doi:10.3389/fmolb.2025.1628118)
Supplement: Supplementary file 2 [file DataSheet1.pdf]

This website uses cookies. By continuing to browse this site, you are agreeing to the use of our site cookies. To find out more, see our Terms of Use. OK

Services | Research | Training | About us

PDBsum entry cdz4

Go to PDB code: cdz4 go

Top page | Protein | Clefts | Pores | Tunnels

Maturase k PDB id **cdz4**

Generate full PROCHECK analyses

PROCHECK summary for cdz4

Contents
Protein chain
A 506 a.a.

Ramachandran plot

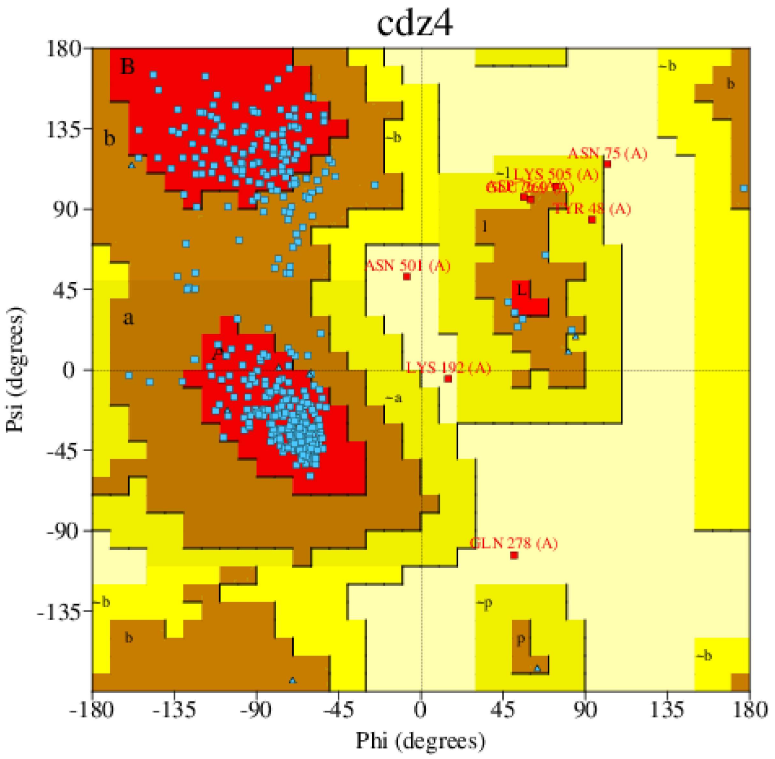

PROCHECK statistics

1. Ramachandran Plot statistics

		No. of residues	%-tage
Most favoured regions	[A,B,L]	415	86.5%*
Additional allowed regions	[a,b,l,p]	57	11.9%
Generously allowed regions	[~a,~b,~l,~p]	4	0.8%
Disallowed regions	[XX]	4	0.8%*
Non-glycine and non-proline residues		480	100.0%
End-residues (excl. Gly and Pro)		2	
Glycine residues		10	
Proline residues		14	
Total number of residues		506	

Based on an analysis of **118** structures of resolution of at least **2.0** Angstroms and *R*-factor no greater than **20.0** a good quality model would be expected to have over **90%** in the most favoured regions [A,B,L].

2. G-Factors

```
                                                Average
Parameter                              Score     Score
---------                              -----     -----
Dihedral angles:-
     Phi-psi distribution              -0.17
     Chi1-chi2 distribution             0.41
     Chi1 only                          0.15
     Chi3 & chi4                        0.83
     Omega                             -0.64*
                                                 -0.05
                                                 =====
Main-chain covalent forces:-
     Main-chain bond lengths            0.54
     Main-chain bond angles            -0.08
                                                  0.18
                                                 =====

     OVERALL AVERAGE                              0.05
                                                 =====
```

G-factors provide a measure of how **unusual**, or out-of-the-ordinary, a property is.

Values below -0.5* - unusual
Values below **-1.0**** - highly unusual

Important note: The main-chain bond-lengths and bond angles are compared with the Engh & Huber (1991) ideal values derived from small-molecule data. Therefore, structures refined using different restraints may show apparently large deviations from normality.

EMBL-EBI

News
Our impact
Contact us
Intranet

Services
By topic
By name (A-Z)
Help & Support

Research
Overview
Publications
Research groups
Postdocs & PhDs

Training
Overview
Live training
On-demand training
Support for trainers
Contact organisers

Industry
Overview
Members Area
Workshops
SME Forum
Contact Industry programme

About us
Overview
Leadership
Funding
Background
Collaboration
Jobs
People & groups
News
Events
Visit us
Contact us

EMBL-EBI, Wellcome Genome Campus, Hinxton, Cambridgeshire, CB10 1SD, UK +44 (0)1223 49 44 44

Copyright © EMBL-EBI 2024 | EBI is an outstation of the European Molecular Biology Laboratory | Terms of use

[back to server]

COFACTOR result for job id MCF14459

[Download result.tar.bz2 for all prediction results]

User Input

>Matk1 (506 residues)
MEKIQRYLQLQRSQQHDFLYPLIFQEYIYVFAHNRVLNRSILSENPGYDNKSSLQIVKRL
ITRMYQQNHFMISSNDSNQNRFWARNNNLYPQIISEGFAFIVEIPFSLQLLSCLEGKKNK
IFKSQNLRSIHSIFPFLEDNFSHLNFVLDILIPHSVHLEILIQTLRYRVKDVSSLHLLRV
FLNQYWSLITPKKVSSSLLKRNQRFFFFLYNSHVCEYDSIFVFLRNQSFHLRSTSSRVLL
ERIYFYIKIERLVNVFVKDFQVNLRFVEEPFMHYIRYQRKSILASKGTSLFMNKWKLYLV
TFWQWHFFVWFRPKRICINQFSKHSLEIFGYLSNVQMSPSVVRSQILENAFLINNAIKKL
DTLVPIIPLIAKLAKAEFCNVLGHPISKPIWADLSDSNIIDRFGRICRNISHYHSGSSNK
KSFYRIKYILRLSCARTLARKHKSTVRAFLKRLGSDLLEEFVMSEEDVLFLTFQKASSAL
RGVYRSRIWYLDMISINDLANLRSKL

Download query sequence and structure

Proteins with similar structure

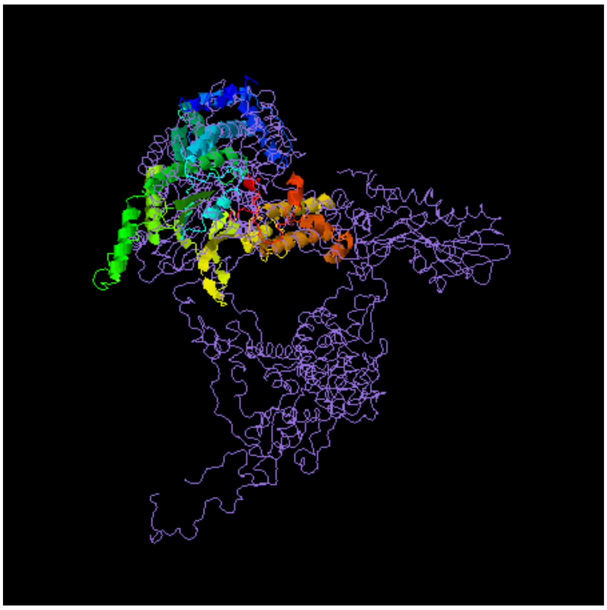

Top 10 structural analogs in PDB (as identified by TM-align)

Click to view	Rank	PDB Hit	TM-score	RMSD[a]	IDEN[a]	Cov.	Download Alignment
○	1	8q91A	0.61	4.98	0.081	0.775	Download
○	2	7qtta	0.61	4.88	0.082	0.769	Download
○	3	8q7nA	0.61	5.03	0.083	0.777	Download
○	4	3jb9A	0.61	4.99	0.084	0.773	Download
○	5	5nrlA	0.60	5.08	0.088	0.773	Download
○	6	4i43B	0.60	5.04	0.091	0.767	Download
○	7	5g2xC	0.58	4.93	0.119	0.727	Download
○	8	2w92A	0.28	7.99	0.040	0.480	Download
○	9	1pciA	0.22	5.96	0.045	0.314	Download
○	10	2wpvC	0.22	6.12	0.071	0.316	Download

(a) Query structure is shown in cartoon, while the structural analog is displayed using backbone trace.
(b) Ranking of proteins is based on TM-score of the structural alignment between the query structure and known structures in the PDB library.
(c) RMSD[a] is the RMSD between residues that are structurally aligned by TM-align.
(d) IDEN[a] is the percentage sequence identity in the structurally aligned region.
(e) Cov. represents the coverage of the alignment by TM-align and is equal to the number of structurally aligned residues divided by length of the query protein.

☐ Spin On/Off

Predicted Gene Ontology (GO) Terms

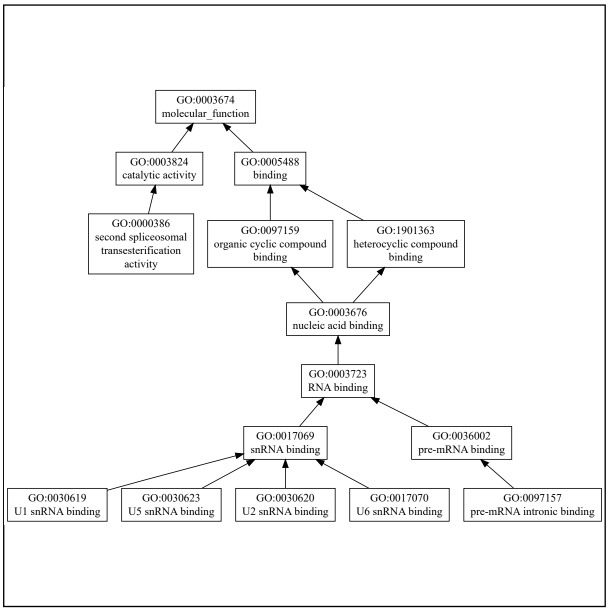

Molecular Function (MF)

GO term	Cscore^{GO}	Name
GO:0097157	0.13	pre-mRNA intronic binding
GO:0030623	0.13	U5 snRNA binding
GO:0030620	0.13	U2 snRNA binding
GO:0030619	0.13	U1 snRNA binding
GO:0017070	0.13	U6 snRNA binding
GO:0000386	0.13	second spliceosomal transesterification activity

Download full result of the above consensus prediction.

Click the graph to show a high resolution version.

(a) Cscore^{GO} is the confidence score of predicted GO terms. Cscore^{GO} values range in between [0-1]; where a higher value indicates a better confidence in predicting the function using the template.
(b) The graph shows the predicted terms within the Gene Ontology hierachy for Molecular Function. Confidently predicted terms are color coded by Cscore^{GO}:

[0.4,0.5) [0.5,0.6) [0.6,0.7) [0.7,0.8) [0.8,0.9) [0.9,1.0]

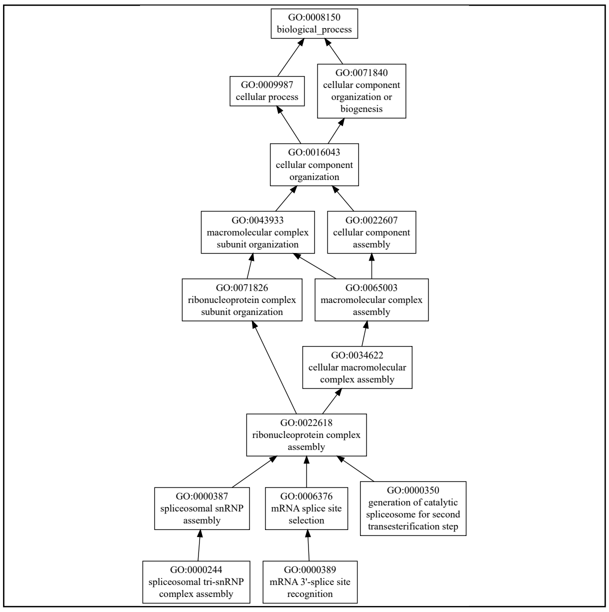

Biological Process (BP)

GO term	Cscore^{GO}	Name
GO:0000389	0.13	mRNA 3'-splice site recognition
GO:0000350	0.13	generation of catalytic spliceosome for second transesterification step
GO:0000244	0.13	spliceosomal tri-snRNP complex assembly

Download full result of the above consensus prediction.

Click the graph to show a high resolution version.

(a) Cscore^{GO} is the confidence score of predicted GO terms. Cscore^{GO} values range in between [0-1]; where a higher value indicates a better confidence in predicting the function using the template.

(b) The graph shows the predicted terms within the Gene Ontology hierachy for Biological Process. Confidently predicted terms are color coded by Cscore^{GO}:

[0.4,0.5) [0.5,0.6) [0.6,0.7) [0.7,0.8) [0.8,0.9) [0.9,1.0]

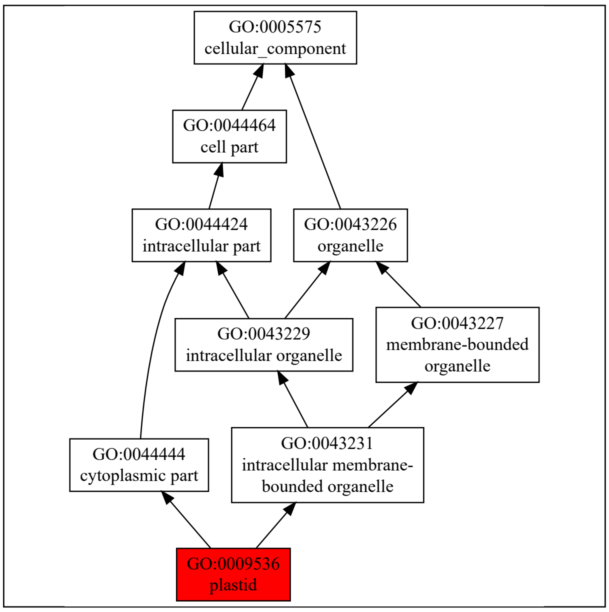

Cellular Component (CC)

GO term	Cscore^{GO}	Name
GO:0009536	1.00	plastid

Download full result of the above consensus prediction.

Click the graph to show a high resolution version.

(a) Cscore^{GO} is the confidence score of predicted GO terms. Cscore^{GO} values range in between [0-1]; where a higher value indicates a better confidence in predicting the function using the template.

(b) The graph shows the predicted terms within the Gene Ontology hierachy for Cellular Component. Confidently predicted terms are color coded by Cscore^{GO}:

[0.4,0.5) [0.5,0.6) [0.6,0.7) [0.7,0.8) [0.8,0.9) [0.9,1.0]

Predicted Enzyme Commission (EC) Numbers

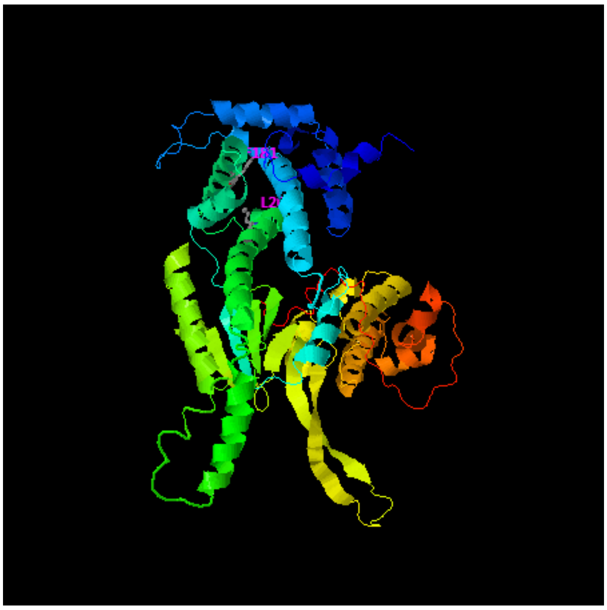

Spin On/Off

Top 5 enzyme homologs in PDB

Click to view	Rank	Cscore^{EC}	PDB Hit	TM-score	RMSD^a	IDEN^a	Cov.	EC Number	Predicted Active Site Residues
○	1	0.060	2vuaA	0.248	6.64	0.056	0.379	3.4.24.69	181,209
○	2	0.060	2hvtB	0.287	6.47	0.056	0.431	2.7.7.49	NA
○	3	0.060	3btaA	0.362	6.93	0.046	0.561	3.4.24.69	NA
○	4	0.060	3mmpG	0.422	6.10	0.084	0.597	2.7.7.48	NA
○	5	0.060	2gv9B	0.358	7.21	0.041	0.569	2.7.7.7	180

Click on the radio buttons to visualize predicted active site residues.

(a) Cscore^{EC} is the confidence score for the Enzyme Commission (EC) number prediction. Cscore^{EC} values range in between [0-1]; where a higher score indicates a more reliable EC number prediction.

(b) TM-score is a measure of global structural similarity between query and template protein.

(c) RMSD^a is the RMSD between residues that are structurally aligned by TM-align.

(d) IDEN^a is the percentage sequence identity in the structurally aligned region.

(e) Cov. represents the coverage of global structural alignment and is equal to the number of structurally aligned residues divided by length of the query protein.

Predicted Ligand Binding Sites

Template proteins with similar binding site:

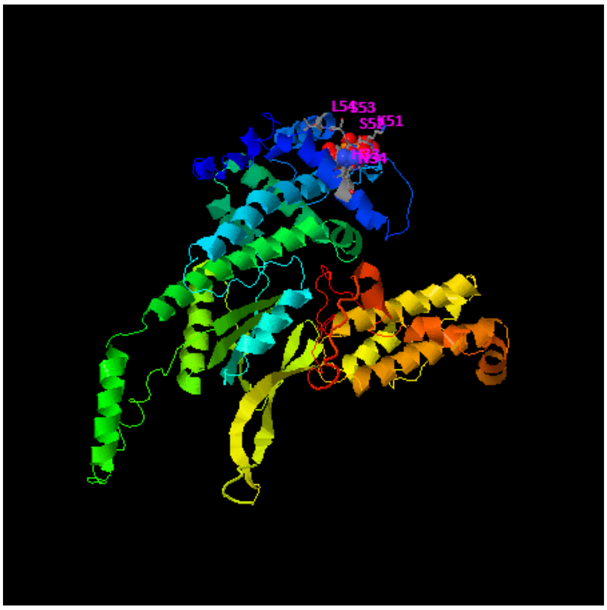

Click to view	Rank	Cscore^{LB}	PDB Hit	TM-score	RMSD^a	IDEN^a	Cov.	BS-score	Lig. Name	Download Complex	Predicted bin[illegible] site residues
○	1	0.02	1n38A	0.522	5.54	0.060	0.692	0.67	CH1	Download	33,34,51,52,53
○	2	0.01	2wrmA	0.489	5.87	0.049	0.672	0.64	QQ3	Download	160,161,209,2[illegible]
○	3	0.01	2wrmA	0.489	5.87	0.049	0.672	0.42	QQ3	Download	54,56,57,60,92

Click on the radio buttons to visualize predicted binding site and residues.

(a) Cscore^{LB} is the confidence score of predicted binding site. Cscore^{LB} values range in between [0-1]; w[illegible] higher score indicates a more reliable ligand-binding site prediction.

(b) BS-score is a measure of local similarity (sequence & structure) between template binding site and pr[illegible] binding site in the query structure. Based on large scale benchmarking analysis, we have observed tha[illegible] score >1 reflects a significant local match between the predicted and template binding site.

(c) TM-score is a measure of global structural similarity between query and template protein.

(d) RMSD^a the RMSD between residues that are structurally aligned by TM-align.

(e) IDEN^a is the percentage sequence identity in the structurally aligned region.

(f) Cov. represents the coverage of global structural alignment and is equal to the number of structurally [illegible] residues divided by length of the query protein.

[Download result.tar.bz2 for all prediction results]

Reference:

- Chengxin Zhang, Peter L Freddolino, and Yang Zhang. COFACTOR: Improved protein function prediction by combining structure, sequence, and protein-protein interaction information. Nucleic Acids Research, 45: W291-299 (2017)
- Ambrish Roy, Jianyi Yang, and Yang Zhang. COFACTOR: An accurate comparative algorithm for structure-based protein function annotation. Nucleic Acids Research, 40:W471-W477 (2012).

[back to server]

ProSA-web
Protein Structure Analysis

Please upload a structure in PDB format: HELP

选择文件 未选择任何文件

Alternatively you can specify a structure by entering its PDB code, chain identifier and NMR model number:

PDB CODE:

PDB CHAIN ID:

PDB MODEL NUMBER:

If you leave the fields for chain id or model number blank, the first chain of the first model found in the PDB file will be analysed.

Analyse

Results for AF-Matk1.pdb, chain A, model 1 (506 aa)

Overall model quality

HELP

Z-Score: **-5.39**

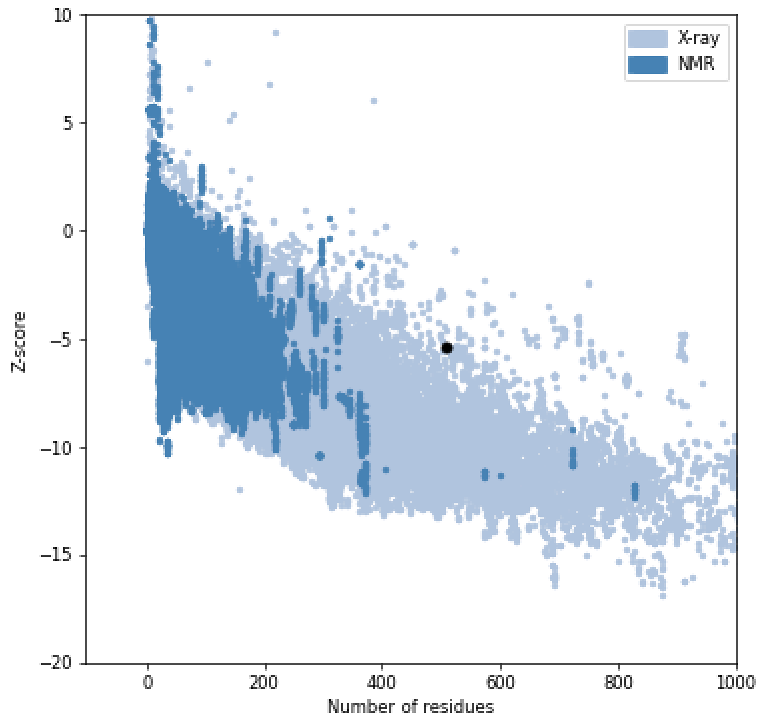

Local model quality

HELP
PNG

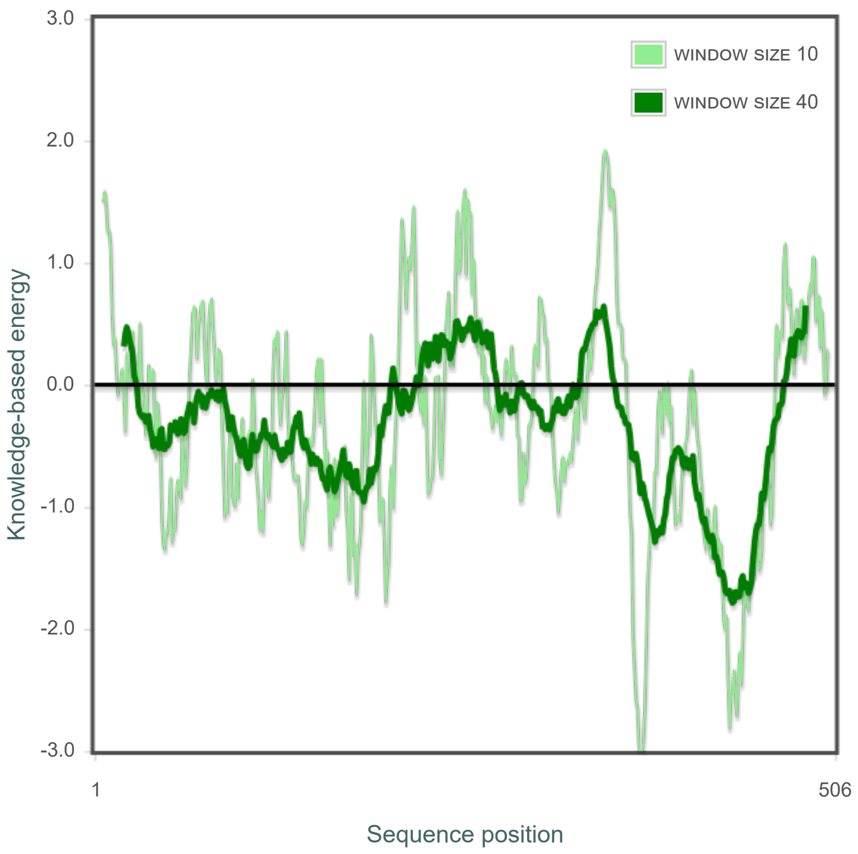

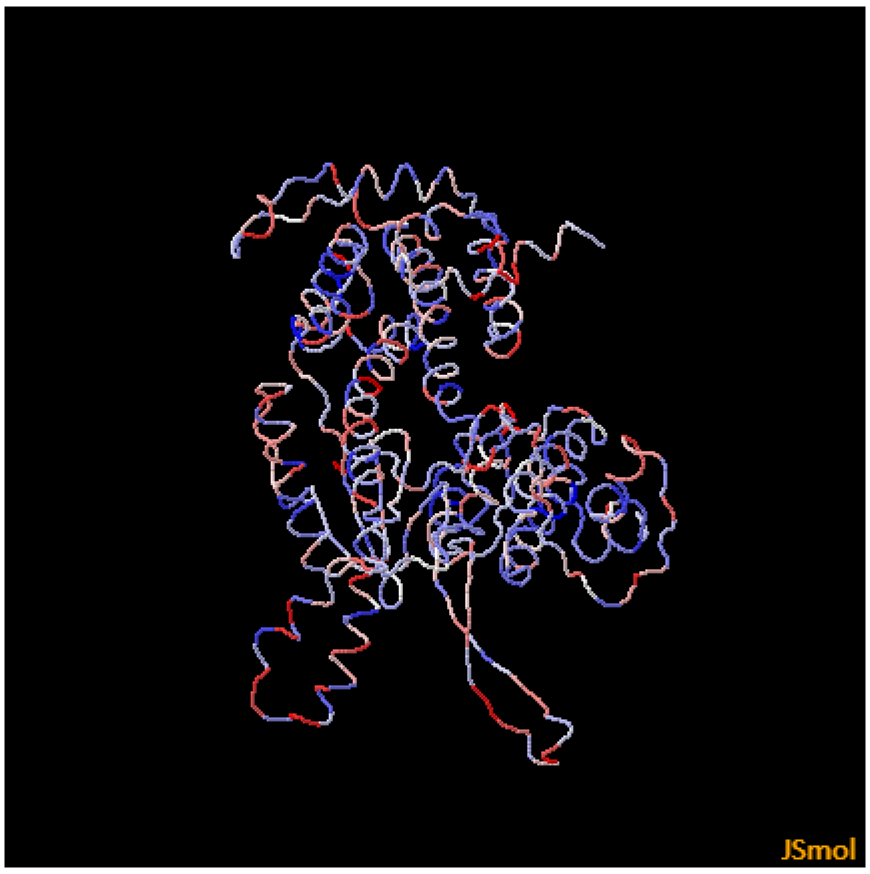

Please cite the following articles if you publish results using ProSA-web:

- Wiederstein & Sippl (2007)
  ProSA-web: interactive web service for the recognition of errors in three-dimensional structures of proteins.
  Nucleic Acids Research 35, W407-W410. [view]
- Sippl, M.J. (1993)
  Recognition of Errors in Three-Dimensional Structures of Proteins.
  Proteins 17, 355-362. [view]

This site is maintained by Markus Wiederstein. For comments and suggestions please contact prosa@came.sbg.ac.at.

The PSIPRED Workbench is undergoing some server upgrades. We apologise for any service disruption. If you experience any issues please email psipred-help@cs.ucl.ac.uk

Name : Matk1

Copy Link: http://bioinf.cs.ucl.ac.uk//psipı

Sequence Plot

Show psipred

Show memsat

Show aatypes

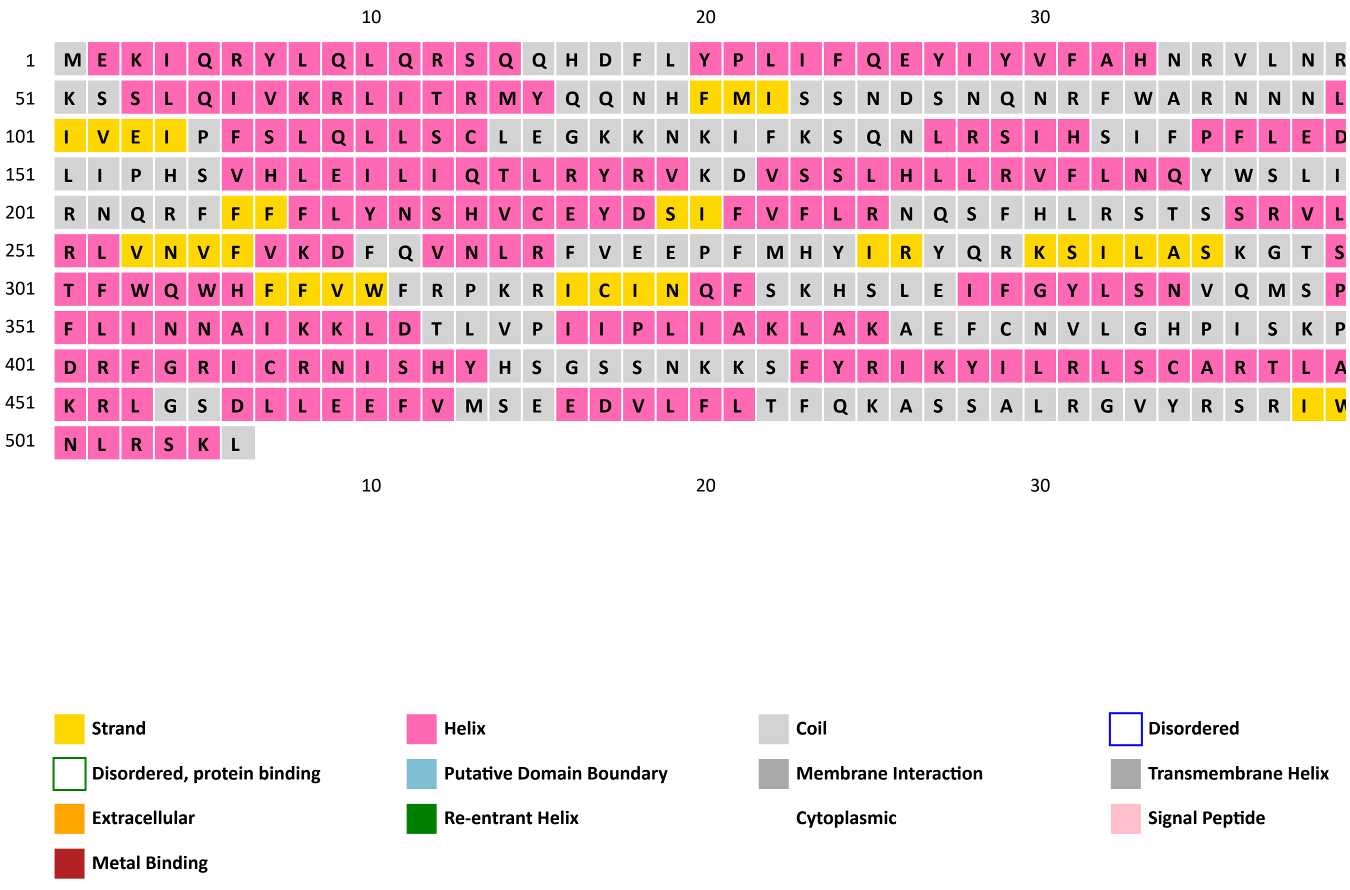

PSIPRED Cartoon

Downloads

RESULTS ZIP FILE

Get Zip File

JOB CONFIGURATION

Get Job Details

PSIPRED DOWNLOADS

Horiz Format Output
SS2 Format Output

Segment Resubmission

+

NPS@: Network Protein Sequence @nalysis

This site is a fork of the original PRABI NPS@ server

[HOME] [DESCRIPTION] [HELP] [NEWS] [CONTACT] [Geno3D]

July 30, 2024: **NPS@** updated (see NEWS).

In your publication cite :
NPS@: Network Protein Sequence Analysis
TIBS 2000 March Vol. 25, No 3 [291]:147-150
Combet C., Blanchet C., Geourjon C. and Deléage G.

Job **SOPMA** (ID: c3808c1308dd) submitted to **NPS@** server on 20250331-183931.

Queued.! Running.! Computed in 32 s !

SOPMA result for : Matk1x0

Abstract Geourjon, C. & Deléage, G., SOPMA: Significant improvement in protein secondary structure prediction by consensus prediction from multiple alignments. Cabios, 1995, 11, 681-684.

```
       10        20        30        40        50        60        70
        |         |         |         |         |         |         |
MEKIQRYLQLQRSQQHDFLYPLIFQEYIYVFAHNRVLNRSILSENPGYDNKSSLQIVKRLITRMYQQNHF
hhhhhhhhhhccccchhhhhhhhhcchhhhhhhhcccccccehhccccccccchhhhhhhhhhhhccccc
MISSNDSNQNRFWARNNNLYPQIISEGFAFIVEIPFSLQLLSCLEGKKNKIFKSQNLRSIHSIFPFLEDN
cccccceecccccccccccccchhhhhhhhhhhhhhhcccccccccccccccccccccccccchhhhhhhhhc
FSHLNFVLDILIPHSVHLEILIQTLRYRVKDVSSLHLLRVFLNQYWSLITPKKVSSSLLKRNQRFFFFLY
cccceeeeecccccccchhhhhhhhhhhcccchhhhhhhhhhhccccceeccccccccccccceechhhh
NSHVCEYDSIFVFLRNQSFHLRSTSSRVLLERIYFYIKIERLVNVFVKDFQVNLRFVEEPFMHYIRYQRK
hhhhhhhhhhhhhhhhhhcccccccccccccccchhhhhhhhhhhhhcchhhhhhhhhhhhheeeeecccc
SILASKGTSLFMNKWKLYLVTFWQWHFFVWFRPKRICINQFSKHSLEIFGYLSNVQMSPSVVRSQILENA
eeeeecccchhhhhhhhhhhhhhhhhhheehccccheeeecccccheeeeeeeecccccceeecccccccc
FLINNAIKKLDTLVPIIPLIAKLAKAEFCNVLGHPISKPIWADLSDSNIIDRFGRICRNISHYHSGSSNK
cccccccccceeeecchhhhhhhhhhhcccccccccccccceeecccchhhhhhhhhhhhhhhhhhhhhhccc
KSFYRIKYILRLSCARTLARKHKSTVRAFLKRLGSDLLEEFVMSEEDVLFLTFQKASSALRGVYRSRIWY
hhhhhhhhhhhhhhhhhhhhhcchhhhhhhhhcccccccccccccchheeeccccccccheccccccceee
LDMISINDLANLRSKL
eehcccchhhhhhhcc
```

Sequence length : 506

SOPMA :
- Alpha helix (Hh) : 227 is 44.86%
- 3_{10} helix (Gg) : 0 is 0.00%
- Pi helix (Ii) : 0 is 0.00%
- Beta bridge (Bb) : 0 is 0.00%
- Extended strand (Ee) : 55 is 10.87%
- Beta turn (Tt) : 0 is 0.00%
- Bend region (Ss) : 0 is 0.00%
- Random coil (Cc) : 224 is 44.27%
- Ambiguous states (?) : 0 is 0.00%
- Other states : 0 is 0.00%

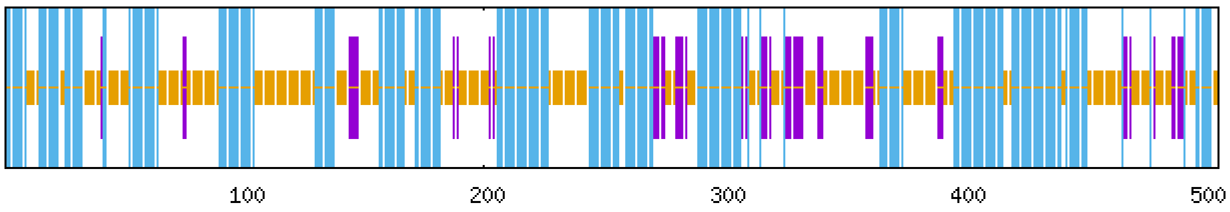

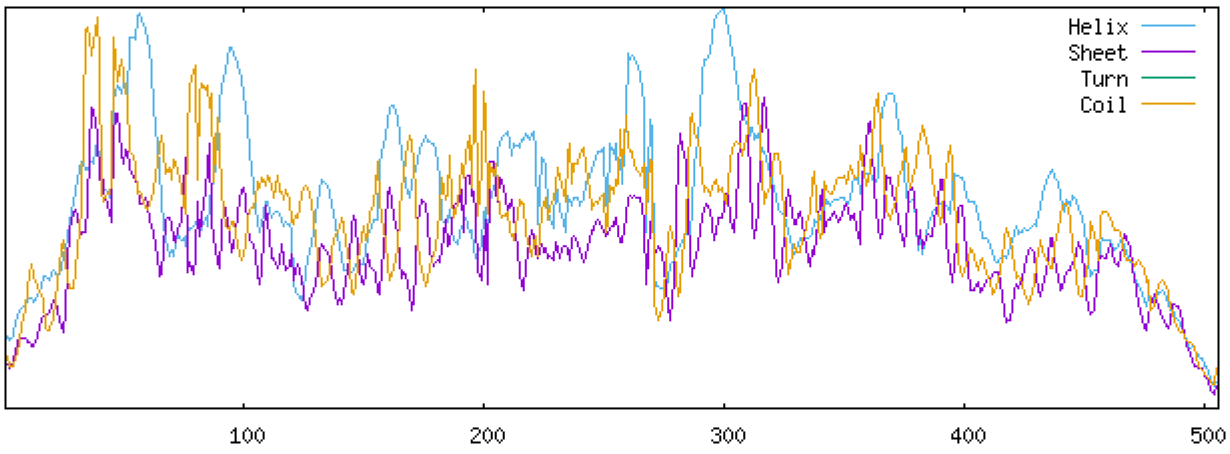

Parameters :
- Window width : 17
- Similarity threshold : 8
- Number of states : 3

Prediction result file (text): [SOPMA]
Intermediate result files (text): [PSI-BLAST on UniProtKB 50% identity] [KALIGN MSA in CLUSTAL W format]

***Last modification time :** Mon Mar 31 18:40:04 2025. **Current time :** Mon Mar 31 18:40:04 2025.* **User :** public@43.242.154.76.

© 1998-2025 Legal notice

This website uses cookies. By continuing to browse this site, you are agreeing to the use of our site cookies. To find out more, see our Terms of Use. OK

PDBsum entry cdz5

Generate full PROCHECK analyses

PROCHECK summary for cdz5

Contents
Protein chain
A 516 a.a.

Ramachandran plot

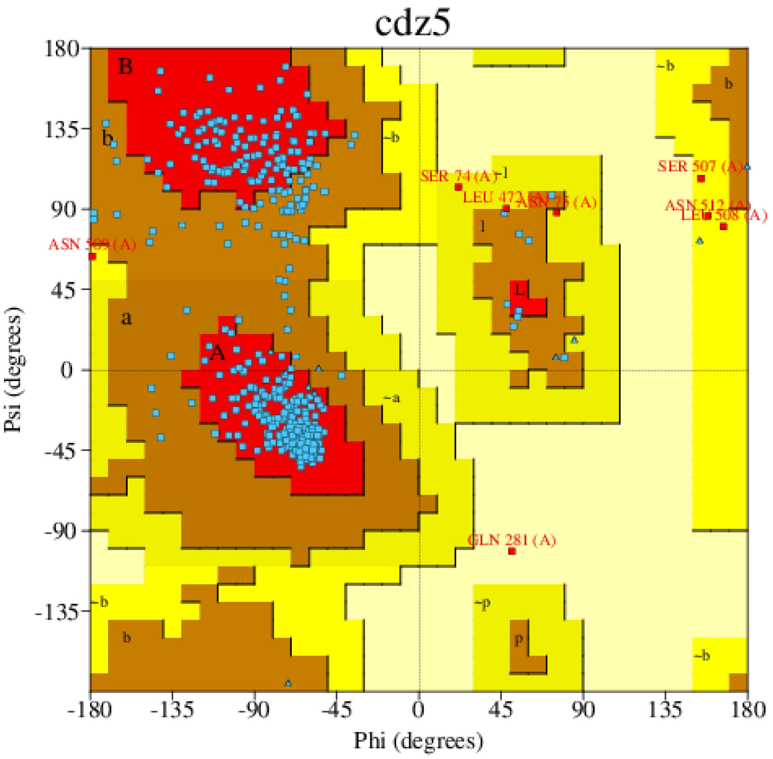

PROCHECK statistics

1. Ramachandran Plot statistics

		No. of residues	%-tage
Most favoured regions	[A,B,L]	413	84.6%*
Additional allowed regions	[a,b,l,p]	67	13.7%
Generously allowed regions	[~a,~b,~l,~p]	7	1.4%
Disallowed regions	[XX]	1	0.2%*
Non-glycine and non-proline residues		488	100.0%
End-residues (excl. Gly and Pro)		2	
Glycine residues		13	
Proline residues		13	
Total number of residues		516	

Based on an analysis of **118** structures of resolution of at least **2.0** Angstroms and *R*-factor no greater than **20.0** a good quality model would be expected to have over **90%** in the most favoured regions [A,B,L].

2. G-Factors

```
                                                   Average
Parameter                              Score       Score
---------                              -----       -----
Dihedral angles:-
     Phi-psi distribution              -0.22
     Chi1-chi2 distribution             0.34
     Chi1 only                          0.26
     Chi3 & chi4                        0.79
     Omega                             -0.77*
                                                   -0.13
                                                   =====
Main-chain covalent forces:-
     Main-chain bond lengths            0.54
     Main-chain bond angles            -0.06
                                                    0.19
                                                   =====

     OVERALL AVERAGE                                0.01
                                                   =====
```

G-factors provide a measure of how **unusual**, or out-of-the-ordinary, a property is.

```
Values below -0.5*  - unusual
Values below -1.0** - highly unusual
```

Important note: The main-chain bond-lengths and bond angles are compared with the Engh & Huber (1991) ideal values derived from small-molecule data. Therefore, structures refined using different restraints may show apparently large deviations from normality.

EMBL-EBI

News
Our impact
Contact us
Intranet

Services
By topic
By name (A-Z)
Help & Support

Research
Overview
Publications
Research groups
Postdocs & PhDs

Training
Overview
Live training
On-demand training
Support for trainers
Contact organisers

Industry
Overview
Members Area
Workshops
SME Forum
Contact Industry programme

About us
Overview
Leadership
Funding
Background
Collaboration
Jobs
People & groups
News
Events
Visit us
Contact us

EMBL-EBI, Wellcome Genome Campus, Hinxton, Cambridgeshire, CB10 1SD, UK +44 (0)1223 49 44 44

Copyright © EMBL-EBI 2024 | EBI is an outstation of the European Molecular Biology Laboratory | Terms of use

[back to server]

COFACTOR result for job id MCF14462

[Download result.tar.bz2 for all prediction results]

User Input

>Matk2 (516 residues)
MEKIQRSLQLKRSQQHDFLYPLIFQEYIYVFAHNRALNRSILSENLGYDNKSSLRIVKRL
ITRMYQQNHFIISSNDSNKNPFLARNTNLYSQIISEGFAFIVEIPFFLQLISCLEGKKNK
IVKSHNLRSIHSIFPFLEDNFSHLNFVVDILIPRSVHAEILIQTLRHWVKDVSSLHLLRV
FLNEYWNWSSLLSPKKVSFSLSKRNQRLFFFLYNSHVCEYESFFTFLRNQSFHLRSTSSG
VLLERIYFYIKIEGLMNTFVKNFQANLGLVEEPCMHYIRYQRKSILASKGTSLFMNKWKF
YLVTFWQWHFSVWFHPRRIWINQFSKHSLSILGYISNVQINPSVVRSQILENAFLINNSI
KKLDTLVPIIPLISELAKAKFCNVFGHPISKPIRADLSDSNIIDRFARICRNISHYHSGS
SKKKSLYRIKYILRLSCARTLARKHKSTVRAFLKRLGSELLEEFLMSEEDVLFLTFQKAA
SAFRGVYRSRIWYLDMISVNDLANQKSLNLGNRNVS

Download query sequence and structure

Proteins with similar structure

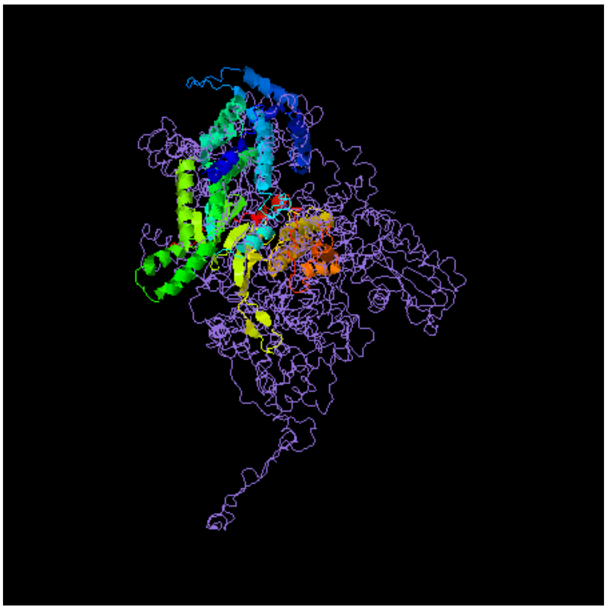

Spin On/Off

Top 10 structural analogs in PDB (as identified by TM-align)

Click to view	Rank	PDB Hit	TM-score	RMSD[a]	IDEN[a]	Cov.	Download Alignment
◯	1	6zymA	0.61	5.35	0.079	0.793	Download
◯	2	7qtta2	0.61	5.26	0.082	0.789	Download
◯	3	5xjcA	0.61	5.26	0.071	0.787	Download
◯	4	3jb9A	0.61	5.32	0.079	0.789	Download
◯	5	4i43B	0.60	5.24	0.073	0.779	Download
◯	6	5ganA	0.60	5.35	0.071	0.783	Download
◯	7	5g2xC	0.57	5.07	0.121	0.729	Download
◯	8	2w92A	0.26	7.44	0.033	0.424	Download
◯	9	2wpvC	0.21	5.73	0.065	0.291	Download
◯	10	1hdhA1	0.21	7.11	0.048	0.333	Download

(a) Query structure is shown in cartoon, while the structural analog is displayed using backbone trace.
(b) Ranking of proteins is based on TM-score of the structural alignment between the query structure and known structures in the PDB library.
(c) RMSD[a] is the RMSD between residues that are structurally aligned by TM-align.
(d) IDEN[a] is the percentage sequence identity in the structurally aligned region.
(e) Cov. represents the coverage of the alignment by TM-align and is equal to the number of structurally aligned residues divided by length of the query protein.

Predicted Gene Ontology (GO) Terms

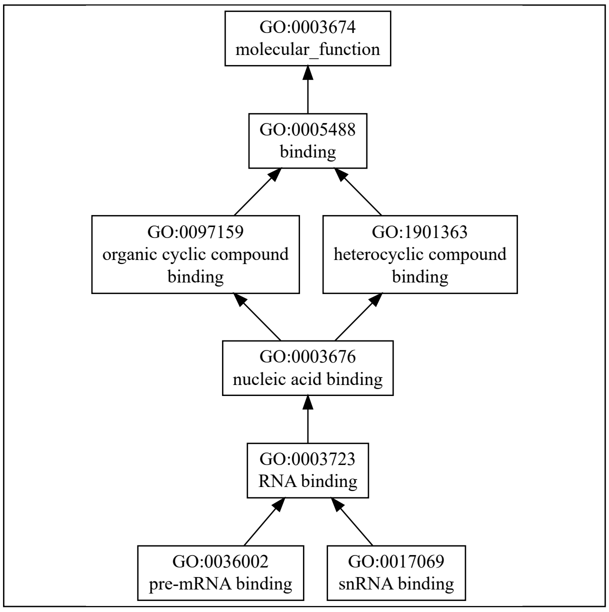

Molecular Function (MF)

GO term	Cscore^{GO}	Name
GO:0036002	0.37	pre-mRNA binding
GO:0017069	0.37	snRNA binding

Download full result of the above consensus prediction.

Click the graph to show a high resolution version.

(a) Cscore^{GO} is the confidence score of predicted GO terms. Cscore^{GO} values range in between [0-1]; where a higher value indicates a better confidence in predicting the function using the template.
(b) The graph shows the predicted terms within the Gene Ontology hierachy for Molecular Function. Confidently predicted terms are color coded by Cscore^{GO}:

[0.4,0.5) [0.5,0.6) [0.6,0.7) [0.7,0.8) [0.8,0.9) [0.9,1.0]

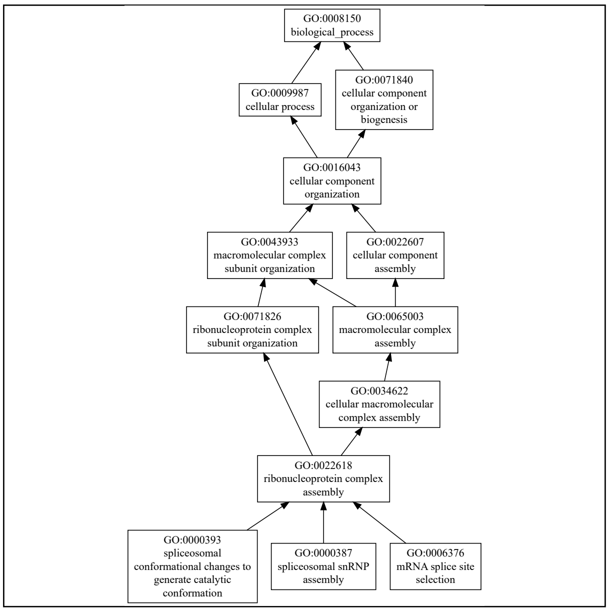

Biological Process (BP)

GO term	Cscore^{GO}	Name
GO:0006376	0.37	mRNA splice site selection
GO:0000393	0.37	spliceosomal conformational changes to generate catalytic conformation
GO:0000387	0.37	spliceosomal snRNP assembly

Download full result of the above consensus prediction.

Click the graph to show a high resolution version.

(a) Cscore^{GO} is the confidence score of predicted GO terms. Cscore^{GO} values range in between [0-1]; where a higher value indicates a better confidence in predicting the function using the template.

(b) The graph shows the predicted terms within the Gene Ontology hierachy for Biological Process. Confidently predicted terms are color coded by Cscore^{GO}:

[0.4,0.5) [0.5,0.6) [0.6,0.7) [0.7,0.8) [0.8,0.9) [0.9,1.0]

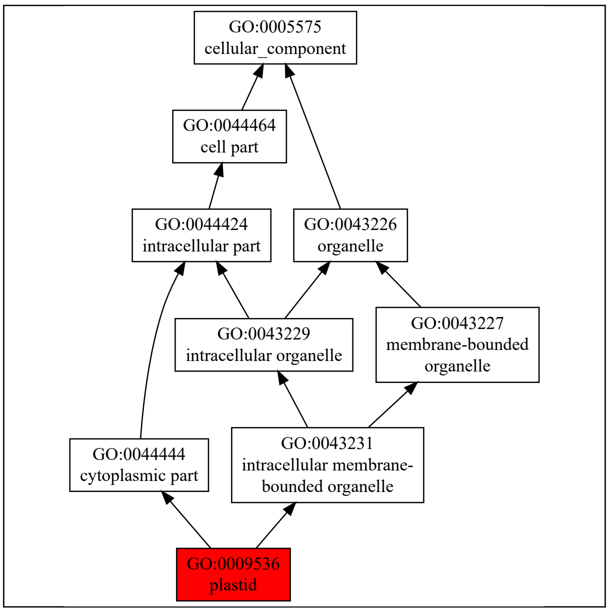

Cellular Component (CC)

GO term	Cscore^{GO}	Name
GO:0009536	1.00	plastid

Download full result of the above consensus prediction.

Click the graph to show a high resolution version.

(a) Cscore^{GO} is the confidence score of predicted GO terms. Cscore^{GO} values range in between [0-1]; where a higher value indicates a better confidence in predicting the function using the template.

(b) The graph shows the predicted terms within the Gene Ontology hierachy for Cellular Component. Confidently predicted terms are color coded by Cscore^{GO}:

[0.4,0.5) [0.5,0.6) [0.6,0.7) [0.7,0.8) [0.8,0.9) [0.9,1.0]

Predicted Enzyme Commission (EC) Numbers

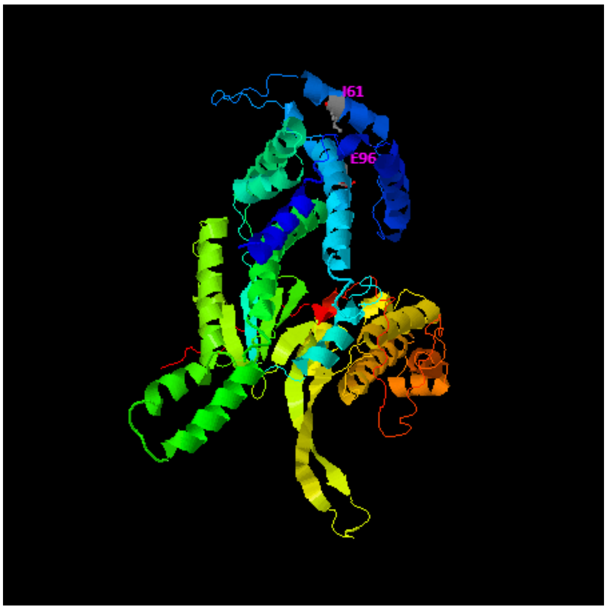

Spin On/Off

Top 5 enzyme homologs in PDB

Click to view	Rank	Cscore^{EC}	PDB Hit	TM-score	RMSD^a	IDEN^a	Cov.	EC Number	Predicted Active Site Residues
○	1	0.060	2vuaA	0.245	6.62	0.038	0.374	3.4.24.69	61,96
○	2	0.060	2hvtB	0.283	6.71	0.055	0.432	2.7.7.49	110
○	3	0.060	3btaA	0.343	7.34	0.047	0.543	3.4.24.69	NA
○	4	0.060	3mmpG	0.427	5.88	0.059	0.595	2.7.7.48	NA
○	5	0.060	1llwA	0.377	7.62	0.056	0.622	1.4.7.1	416

Click on the radio buttons to visualize predicted active site residues.

(a) Cscore^{EC} is the confidence score for the Enzyme Commission (EC) number prediction. Cscore^{EC} values range in between [0-1]; where a higher score indicates a more reliable EC number prediction.

(b) TM-score is a measure of global structural similarity between query and template protein.

(c) RMSD^a is the RMSD between residues that are structurally aligned by TM-align.

(d) IDEN^a is the percentage sequence identity in the structurally aligned region.

(e) Cov. represents the coverage of global structural alignment and is equal to the number of structurally aligned residues divided by length of the query protein.

Predicted Ligand Binding Sites

Template proteins with similar binding site:

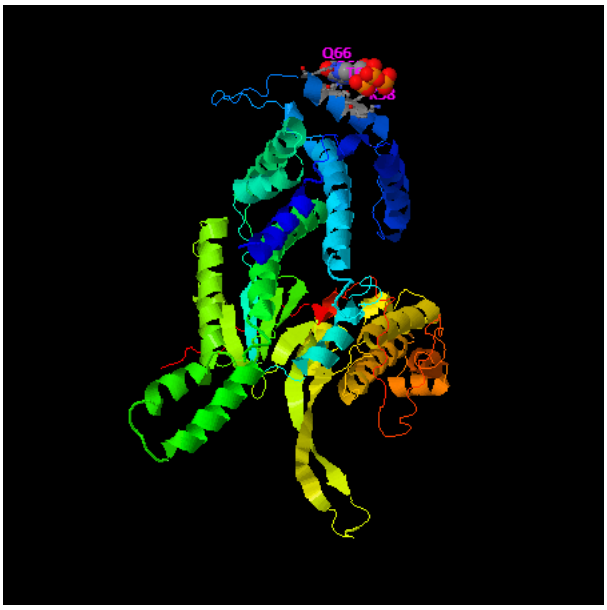

Click to view	Rank	Cscore^{LB}	PDB Hit	TM-score	RMSD^a	IDEN^a	Cov.	BS-score	Lig. Name	Download Complex	Predicted binc
◯	1	0.01	2xi3A	0.496	6.15	0.061	0.704	0.89	GTP	Download	58,59,62,63,66
◯	2	0.01	1n38A	0.517	5.69	0.059	0.692	0.60	CH1	Download	153,154,155,1
◯	3	0.01	3fqlA	0.496	6.23	0.054	0.707	0.44	79Z	Download	137,278,280,2
◯	4	0.01	2wrmA	0.484	5.99	0.059	0.676	0.51	QQ3	Download	159,160,212,2
◯	5	0.01	1n38A	0.517	5.69	0.059	0.692	0.42	QNA	Download	130,131,334
◯	6	0.01	2wrmA	0.484	5.99	0.059	0.676	0.46	QQ3	Download	54,56,57,60,93
◯	7	0.01	3cj5A	0.496	6.04	0.059	0.696	0.42	SX6	Download	22,25,26,27,31

Click on the radio buttons to visualize predicted binding site and residues.

(a) Cscore^{LB} is the confidence score of predicted binding site. Cscore^{LB} values range in between [0- indicates a more reliable ligand-binding site prediction.

(b) BS-score is a measure of local similarity (sequence & structure) between template binding site and pr query structure. Based on large scale benchmarking analysis, we have observed that a BS-score >1 match between the predicted and template binding site.

(c) TM-score is a measure of global structural similarity between query and template protein.

(d) RMSD^a the RMSD between residues that are structurally aligned by TM-align.

(e) IDEN^a is the percentage sequence identity in the structurally aligned region.

(f) Cov. represents the coverage of global structural alignment and is equal to the number of structurally a length of the query protein.

[Download result.tar.bz2 for all prediction results]

Reference:

- Chengxin Zhang, Peter L Freddolino, and Yang Zhang. COFACTOR: Improved protein function prediction by combining structure, sequence, and protein-protein interaction information. Nucleic Acids Research, 45: W291-299 (2017)
- Ambrish Roy, Jianyi Yang, and Yang Zhang. COFACTOR: An accurate comparative algorithm for structure-based protein function annotation. Nucleic Acids Research, 40:W471-W477 (2012).

[back to server]

ProSA-web
Protein Structure Analysis

Please upload a structure in PDB format: HELP

选择文件 未选择任何文件

Alternatively you can specify a structure by entering its PDB code, chain identifier and NMR model number:

PDB CODE:

PDB CHAIN ID:

PDB MODEL NUMBER:

If you leave the fields for chain id or model number blank, the first chain of the first model found in the PDB file will be analysed.

Analyse

Results for AF-Matk2.pdb, chain A, model 1 (516 aa)

Overall model quality

HELP

Z-Score: **-5.68**

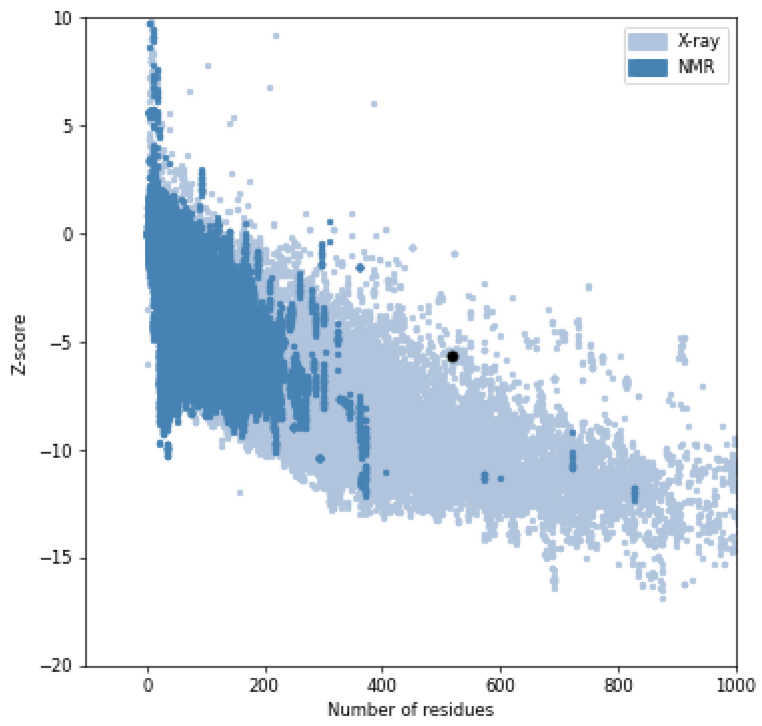

Local model quality

HELP
PNG

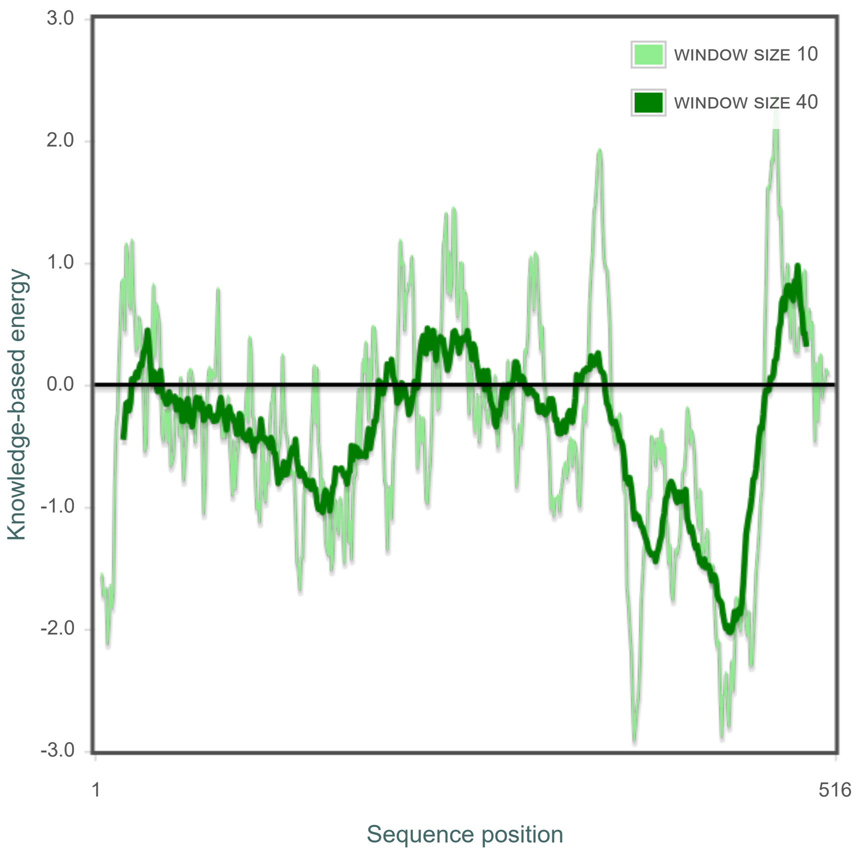

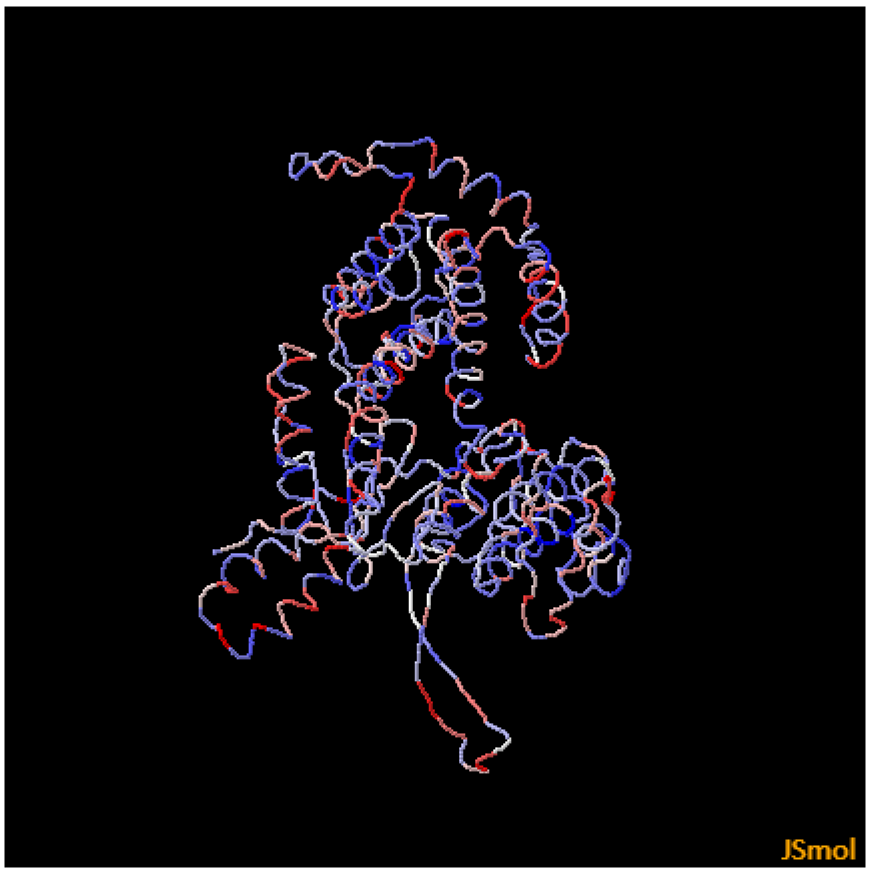

Please cite the following articles if you publish results using ProSA-web:

- Wiederstein & Sippl (2007)
  ProSA-web: interactive web service for the recognition of errors in three-dimensional structures of proteins.
  Nucleic Acids Research 35, W407-W410. [view]
- Sippl, M.J. (1993)
  Recognition of Errors in Three-Dimensional Structures of Proteins.
  Proteins 17, 355-362. [view]

This site is maintained by Markus Wiederstein. For comments and suggestions please contact prosa@came.sbg.ac.at

The PSIPRED Workbench is undergoing some server upgrades. We apologise for any service disruption. If you experience any issues please email psipred-help@cs.ucl.ac.uk

Name : Matk2

Copy Link: http://bioinf.cs.ucl.ac.uk//psipı

Sequence Plot

Show psipred | Show memsat | Show aatypes

	10 · 20 · 30
1	M E K I Q R S L Q L K R S Q Q H D F L Y P L I F Q E Y I Y V F A H N R A L N R
51	K S S L R I V K R L I T R M Y Q Q N H F I I S S N D S N K N P F L A R N T N L
101	I V E I P F F L Q L I S C L E G K K N K I V K S H N L R S I H S I F P F L E D
151	L I P R S V H A E I L I Q T L R H W V K D V S S L H L L R V F L N E Y W N W S
201	L S K R N Q R L F F F L Y N S H V C E Y E S F F T F L R N Q S F H L R S T S S
251	K I E G L M N T F V K N F Q A N L G L V E E P C M H Y I R Y Q R K S I L A S K
301	Y L V T F W Q W H F S V W F H P R R I W I N Q F S K H S L S I L G Y I S N V Q
351	E N A F L I N N S I K K L D T L V P I I P L I S E L A K A K F C N V F G H P I
401	N I I D R F A R I C R N I S H Y H S G S S K K K S L Y R I K Y I L R L S C A R
451	A F L K R L G S E L L E E F L M S E E D V L F L T F Q K A A S A F R G V Y R S
501	D L A N Q K S L N L G N R N V S

10 · 20 · 30

Strand
Helix
Coil
Disordered
Disordered, protein binding
Putative Domain Boundary
Membrane Interaction
Transmembrane Helix
Extracellular
Re-entrant Helix
Cytoplasmic
Signal Peptide
Metal Binding

PSIPRED Cartoon

Downloads

RESULTS ZIP FILE

Get Zip File

JOB CONFIGURATION

Get Job Details

PSIPRED DOWNLOADS

Horiz Format Output
SS2 Format Output

Segment Resubmission

+

NPS@: Network Protein Sequence @nalysis

This site is a fork of the original PRABI NPS@ server

[HOME] [DESCRIPTION] [HELP] [NEWS] [CONTACT] [Geno3D]

July 30, 2024: **NPS@** updated (see NEWS).

In your publication cite :
NPS@: Network Protein Sequence Analysis
TIBS 2000 March Vol. 25, No 3 [291]:147-150
Combet C., Blanchet C., Geourjon C. and Deléage G.

Job **SOPMA** (ID: 9c4ffd546e34) submitted to **NPS@** server on 20250331-184006.

Queued.! Running.! Computed in 30 s !

SOPMA result for : Matk2x0

Abstract Geourjon, C. & Deléage, G., SOPMA: Significant improvement in protein secondary structure prediction by consensus prediction from multiple alignments. Cabios, 1995, 11, 681-684.

```
      10        20        30        40        50        60        70
       |         |         |         |         |         |         |
MEKIQRSLQLKRSQQHDFLYPLIFQEYIYVFAHNRALNRSILSENLGYDNKSSLRIVKRLITRMYQQNHF
hhhhhhhhhhccccchhhhhhhhhcchheeehhhhhhcccccccccccccccchhhhhhhhhhhhccccc
IISSNDSNKNPFLARNTNLYSQIISEGFAFIVEIPFFLQLISCLEGKKNKIVKSHNLRSIHSIFPFLEDN
eeecccccccccccccccccchhhhhhhhhhhhhhhcccccccccccccccccccccccccchhhhhhhhhc
FSHLNFVVDILIPRSVHAEILIQTLRHWVKDVSSLHLLRVFLNEYWNWSSLLSPKKVSFSLSKRNQRLFF
cccceeeeecccccccchhhhhhhhhhhcccchhhhhhhhhhhcccccccccccccccccccccccccch
FLYNSHVCEYESFFTFLRNQSFHLRSTSSGVLLERIYFYIKIEGLMNTFVKNFQANLGLVEEPCMHYIRY
hhhhhhhhhhhhhhhhhhhhhhccccccccccchhhhhhhhhhhhhhhhcccccccccccccceeeeeec
QRKSILASKGTSLFMNKWKFYLVTFWQWHFSVWFHPRRIWINQFSKHSLSILGYISNVQINPSVVRSQIL
ccceeeeecccchhhhhhhhhhhhhhhhhhhheehccccceeeecccccheeeeeeccccccccccccccc
ENAFLINNSIKKLDTLVPIIPLISELAKAKFCNVFGHPISKPIRADLSDSNIIDRFARICRNISHYHSGS
cccccccccccceeeecchhhhhhhhhhccccccccccccccceeecccchhhhhhhhhhhhhhhhhhhhh
SKKKSLYRIKYILRLSCARTLARKHKSTVRAFLKRLGSELLEEFLMSEEDVLFLTFQKAASAFRGVYRSR
ccchhhhhhhhhhhhhhhhhhhhhcchhhhhhhhhccceeeecccccccceeeecccccccccccccccc
IWYLDMISVNDLANQKSLNLGNRNVS
eeeeeeechhhhhhhhhhccccccce
```

Sequence length : 516

SOPMA :
- Alpha helix (Hh) : 216 is 41.86%
- 3_{10} helix (Gg) : 0 is 0.00%
- Pi helix (Ii) : 0 is 0.00%
- Beta bridge (Bb) : 0 is 0.00%
- Extended strand (Ee) : 57 is 11.05%
- Beta turn (Tt) : 0 is 0.00%
- Bend region (Ss) : 0 is 0.00%
- Random coil (Cc) : 243 is 47.09%
- Ambiguous states (?) : 0 is 0.00%
- Other states : 0 is 0.00%

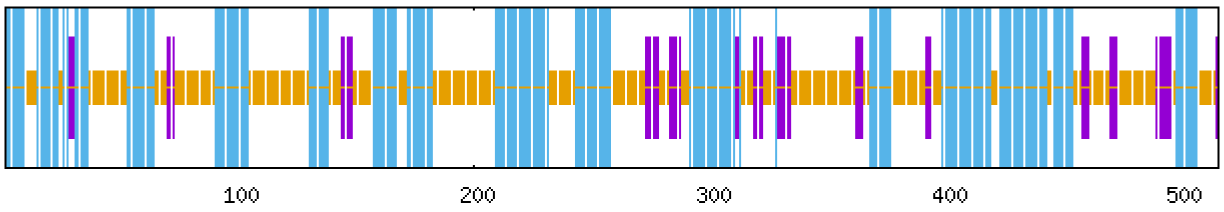

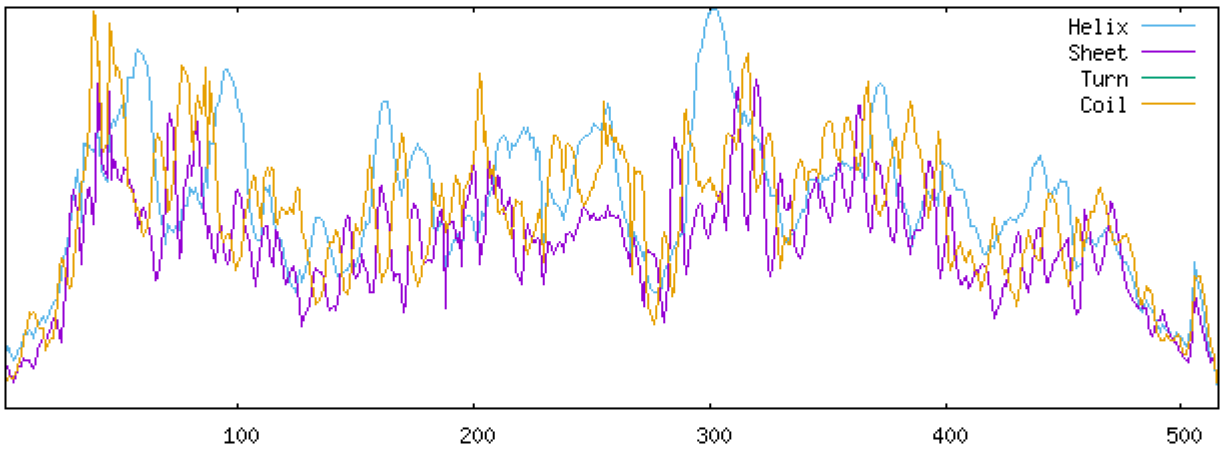

Parameters :
- Window width : 17
- Similarity threshold : 8
- Number of states : 3

Prediction result file (text): [SOPMA]
Intermediate result files (text): [PSI-BLAST on UniProtKB 50% identity] [KALIGN MSA in CLUSTAL W format]

***Last modification time :** Mon Mar 31 18:40:37 2025. **Current time :** Mon Mar 31 18:40:37 2025.* **User :** public@43.242.154.76.

© 1998-2025 Legal notice
